# OLFML3 negatively regulates RIG-I signaling in RNA virus infection

**DOI:** 10.3389/fimmu.2026.1810852

**Published:** 2026-06-10

**Authors:** Qian Gu, Hong Mei, Qijun Yu, Peihong Yu, Junjie Zhang, Pengfei Hu, Jieming Qu, Jia Liu

**Affiliations:** 1 Shanghai Institute for Advanced Immunochemical Studies and School of Life Science and Technology, ShanghaiTech University, Shanghai, China; 2 Department of Pulmonary and Critical Care Medicine, Ruijin Hospital, Institute of Respiratory Diseases, Shanghai Jiao Tong University School of Medicine, Shanghai, China; 3 Guangzhou Laboratory, Guangzhou, Guangdong, China; 4 Shanghai Clinical Research and Trial Center, Shanghai, China

**Keywords:** antiviral immunity, IFN-I signaling, OLFML3, RIG-I, TRIM21

## Abstract

**Background:**

Olfactomedin-like protein 3 (OLFML3) is a secreted glycoprotein that is primarily deemed to be associated with embryonic development, angiogenesis, and tumorigenesis. Recent studies have highlighted the function of OLFML3 as an important regulatory protein in viral and bacterial infections. This study explores the role of OLFML3 in type I interferon (IFN-I) signaling in RNA virus infection and related mechanism of action.

**Methods:**

RT-qPCR was used to determine the effects of OLFML3 on IFN-I production in the RNA virus infection. *Olfml3*^-/-^ mice was used to evaluate the effects of OLFML3 in in vivo viral infection. Western blotting (WB) and immunofluorescence microscropy experiments were conducted to analyze the effects of OLFML3 on retinoic-acid-inducible gene I (RIGI)-mediated IFN-I signaling pathway. Mass spectrometry and co-immunoprecipitation (Co-IP) were used to analyze the partner proteins of OLFML3. Gain-of-function studies by overexpression and loss-of-function studies by gene knockout and knockdown were performed to understand the functions of OLFML3 and TRIM21 in RIG-I-mediated IFN-I signaling pathway.

**Results:**

OLFML3 can inhibit IFN-I production in RNA virus infection by suppressing RIG-I signaling. OLFML3 interacts with the PRY/SPRY domain of TRIM21, an E3 ubiquitinligase. OLFML3 can disrupt TRIM21-mediated RIG-I K63-ubiquitination, leading to the destabilization of RIG-I and suppressed IFN-I signaling.

**Conclusion:**

OLFML3 functions as a general immunosuppressor in RNA virus infection. These results may help developing OLFML3-targeted antiviral therapeutics for the re-activation of IFN-I signaling.

## Introduction

Olfactomedin-like 3 (OLFML3), also known as hOLF44, is a member of the olfactomedin-like (OLF) protein family (1) and belongs to the subfamily VII (2). The human OLFML3 gene, located at chromosome 1p13.1, spans 2.9 kb and comprises three exons and two introns. Its transcript is a 1, 852-nucleotide mRNA containing a 1, 221-nt coding sequence (CDS) flanked by 5′ and 3′ untranslated regions (UTRs) (1). OLFML3 is a secreted glycoprotein composed of 406 amino acids (3). OLFML3 contains a signal peptide at the N-terminus, a coiled-coil domain in the middle, and a C-terminal OLF domain. OLFML3 is differentially expressed in various tissues and highly expressed in placenta (4)^-^ (5), blood vessels, and multiple human cancers (6).

OLFML3 exerts important functions in embryonic development, angiogenesis, and tumor infiltration (7–10). OLFML3, known as ONT1 in *Xenopus*, can function as a scaffold protein and stabilize axial formation during the embryogenesis of *Xenopus* (7). OLFML3 can also act as a proangiogenic factor and promote angiogenesis during embryonic blood vessel formation or in tumor microenvironments (8, 9). Owing to its proangiogenic activity, OLFML3 has been used to facilitate neovascularization during tissue regeneration and shown to be capable of promoting endothelial cell proliferation and migration (11).

In addition, OLFML3 is important in microglia and the tumorigenesis of glioblastoma. In mouse microglia, Olfml3 is co-localized with Iba1, a multi-functional protein essential for microglial activation and migration, and is related with neuroinflammation (12). *Olfml3* expression is transcriptionally regulated by TGF-β/Smad2 signaling (13). In glioblastoma (GBM), OLFML3 is involved in circadian locomotor output cycles kaput (CLOCK)-mediated tumorigenesis (14–17). Targeting OLFML3-mediated signaling appeared to be an efficient approach to treating GBM (18, 19). Besides GBM, OLFML3 is found to be related with colorectal cancer (6) and liver cancer (20), though the mechanism of action is different from that in GBM.

Despite these advances, the biological function of OLFML3 in innate immunity has not been well characterized. In a previous study, we identified OLFML3 as a negative regulator of type I interferon (IFN) signaling (21). A subsequent study showed that OLFML3 was also involved in LPS-induced mitochondrial dysfunction in macrophages (22). In the present study, we investigated the function of OLFML3 in a broader range of RNA viruses and discovered that OLFML3 is a general antagonist to type I IFN signaling during RNA virus infection. We showed that OLFML3 regulated type I IFN production by modulating RIG-I and that the mechanism of action involved TRIM21-mediated K63 ubiquitination of RIG-I.

## Materials and methods

### Animal experiments

*Olfml3*^-/-^ mice on a C57BL/6 background were generated by Gempharmatech Co. (Nanjing, China). The genotyping was performed by PCR reactions using primers (**Supplementary Table 1**) and extracted genomic DNA from tail specimens. All animal experiments complied with ethical regulations and were approved by the ethical committee of ShanghaiTech University (approval numbers 20230821001 and 20250513001).

### Viruses

VSV (NCBI: OP734420.1), influenza virus A/PR8/34(H1N1) (PR8 IAV, NCBI: KC815527.1), HCoV-229E (NCBI: OK662398.1), and HCoV-OC43 (NCBI: PQ630153.1) were kindly provided by Prof. Jincun Zhao (Guangzhou Medical University). VSV was propagated in VeroE6 cells. Vero cells were infected at a low multiplicity of infection (MOI), and the supernatant was collected at 72 h after infection. The titer of VSV was measured using plaque assay as described (23).

### Antibodies and reagents

Antibodies anti-TBK1 (cat. no. 38066S), anti-phospho-TBK1 (cat. no. 5483S), anti-phospho-IRF3 (cat. no. 4947S), anti-MAVS (cat. no. 3993T), and anti-Flag (cat. no. 14793S) were purchased from Cell Signaling Technology (Danvers, MA, USA). Goat anti-rabbit secondary antibody conjugated with Alexa-488 (cat. no. A11029) and goat anti-mouse secondary antibody conjugated with Alexa-568 (cat. no. A11031) were purchased from Invitrogen. Anti-HA (cat. no. AE036) was from Abclonal. Anti-GAPDH (cat. no. HRP-60004-100UL) was from Proteintech Wuhan, China. Anti-OLFML3 antibody (cat. no. PA531581) was from Thermo (Waltham, MA, USA). Anti-TRIM21 antibody (cat. no. ab207728-40ul), anti-ubiquitin (cat. no. ab134953), and anti-IRF3 (cat. no. ab68481) were purchased from Abcam (Cambridge, MA, USA). MG132 (cat. no. S2619) was acquired from Selleck (Houston, TX, USA). Poly(I:C) (HMW) (cat. no. tlrl-pic) was obtained from Invivogen (Toulouse, France).

### Cells and culture

H1-HeLa cells, HEK-293T cells, RAW264.7, and MLE-12 cells were cultured at 37°C under 5% CO_2_ in DMEM (cat. no. D0819, Sigma-Aldrich, St. Louis, MO, USA) supplemented with 10% fetal bovine serum (cat. no. 10091148, Gibco, Gaithersburg, MD, USA) and 1% penicillin and streptomycin (cat. no. 15140122, Gibco). BMDMs were isolated from the tibia and femur of *Olfml3^+/+^* and *Olfml3^-/-^* mice and then cultured in IMDM (cat. no. 12440053, Gibco) with 10% FBS and 20% L929 (cat. no. CCL-1, ATCC) supernatant at 37°C for 7 days, followed by the addition of medium at 3.5 days.

### *In vitro* transcription of 5′ triphosphate RNA

5′ Triphosphate RNA (5′-ppp RNA) RNA was synthesized through a multi-step process starting with the chemical synthesis of complementary single-stranded DNA sequences with the T7 promoter. After synthesis, the single-stranded DNA sequences were annealed and purified to generate double-stranded DNA templates. These double-stranded DNA products were then subjected to *in vitro* transcription using T7 High Yield RNA Transcription Kit (Yeasen, Cat#10623ES50) according to the manufacturer’s protocol. DNase I treatment was applied for 15 min to remove residual DNA templates. The synthesized 5′-ppp RNA was then purified using TRIzol reagent for subsequent applications.

### Plasmid construction and transfection

*OLFML3* and *TRIM21* genes and their truncation constructs were cloned into the pCAGGS vector with indicated tags for transient expression. Mammalian expression plasmids for *RIG-I*, *MDA5*, *MAVS*, *TBK1*, *IRF3*, *IRF7*, and *DDX41* were amplified by standard PCR from THP1 cDNA and then cloned into the pCAGGS vector with HA tag. Renilla luciferase reporter, IFN-β, and ISRE promoter luciferase reporter plasmids were maintained in our laboratory. Flag-Ub, Flag-K27Ub, Flag-K48Ub, and Flag-K63Ub (where all K other than the indicated ones were mutated to R) were synthesized and cloned into pEGFP-C1 vector by Beijing Tsingke Biotech Co. (Beijing, China). All plasmids were confirmed by DNA sequencing. Plasmid transfection was conducted using Lipofectamine 3000 (cat. no. L3000015, Thermo).

### Generation of *OLFML3* knockout cells

*OLFML3*^-/-^ cells were constructed using the CRISPR/Cas 9 system. Pre-synthesized sgRNA (**Supplementary Table 2**) was cloned to lentiCRISPR-v2 vector carrying Cas9 expression sequence. Envelope plasmid psPAX, package plasmid pMD2.G, and LentiCRISPR-v2 were transfected to HEK-293T cells at 90% density to produce lentivirus. At 48 h after transfection, the supernatant containing lentivirus particles was collected by centrifugation at 500 g for 10 min.

Cells at 90% confluency were transduced with lentivirus by spinning at 600 g for 90 min at 37 °C and then selected with 0.25–1 μg/mL puromycin for 5–7 days to eliminate the uninfected cells. Single-cell clones containing sgRNAs were sorted by using Moflo Astrios EQ (Beckman Coulter) and validated by PCR using primers (**Supplementary Table 3**) and Sanger sequencing. The gene knockout efficiency was determined by TIDE analysis of the Sanger sequencing results (https://tide.nki.nl/).

### Luciferase assay

HEK-293T cells at 90% confluency were seeded in a 96-well plate and transfected with 5 ng Renilla luciferase reporter plasmid (internal control), 20 ng IFN-β or ISRE luciferase reporter-encoding plasmid, and a gene-of-interest expression plasmid as appropriate in each well. At 16 h after transfection or stimulation, luciferase assays were performed with the procedures provided by the manufacturer (cat. no. DL101-01, Vazyme, Nanjing, China).

### Enzyme-linked immunosorbent assay

The concentrations of mouse IFN-β and IL-6 in BMDM and RAW264.7 culture supernatants and serum were measured by using ELISA Kits (cat. no. luex-mifnbv2, InvivoGen and cat. no. 1210602, Dakewe Biotech Co, Shanghai, China). The concentration of human IFN-β in H1-HeLa culture supernatant was measured by using ELISA Kits (cat. no. DY814-05, R&D Systems).

### Real-time quantitative PCR

Total RNA was extracted from tissues and cells lysed by using TRIzol reagent (cat. no. 15596018CN, Thermo). Purified RNA was reverse-transcribed to complementary DNA (cDNA) using PrimeScript™ RT reagent Kit with gDNA Eraser (cat. no. RR047A, Takara Dalian, China). The mRNA expression levels were detected by real-time PCR using SYBR Green (cat. no. AG11718, Accurate Biology, Hunan, China) with the primers (**Supplementary Table 4**) listed in the supplementary information on Applied Biosystems Quanut Studio 7 Real-Time PCR System. Target genes were normalized to *β-actin* for human genes or *hprt1* for mouse genes.

### Generation of *OLFML3* stable overexpressing cell lines

Codon-optimized *OLFML3* sequence (**Supplementary Table 5**) was cloned to pLV-IRES-zsGreen vector. Silent mutations were introduced to the protospacer adjacent motif (PAM) and 20-bp sgRNA-targeting sequence. pLV-IRES-zsGreen-OLFML3, envelope plasmid psPAX, and package plasmid pMD2.G were transfected to HEK-293T cells for lentivirus packaging. The target cells were spin-infected with lentivirus as described above and sorted by green fluorescence to obtain *OLFML3*-expressing stable cells.

### Gene knockdown using siRNA

siRNA sequences were designed and synthesized by GenePharma Co (Shanghai, China). HEK-293T cells were seeded onto 12-well plates with 80% density and then were transfected with 20 pmol TRIM21 siRNA (**Supplementary Table 6**) by 2 μL Lipofectamine2000 for 48 h. The expression level of endogenous *TRIM21* was quantified by western blotting (WB).

### Co-immunoprecipitation and WB

Expression plasmids with indicated tags were transfected into HEK-293T cells. At 24 h after transfection, cells were lysed by treatment with IP lysis buffer (cat. no. 87787, Thermo) for 10 min. The lysate was centrifugated at 13, 000 g for 5 min at 4°C. The supernatant was collected as whole cell lysates. The total concentration of proteins in the lysate was measured by using BCA Protein Assay Kit (cat. no. P0012, Beyotime, Shanghai, China). For immunoprecipitation, 30 mg lysate was incubated with 5 μL anti-HA magnetic beads (cat. no. 88836, Thermo) or 10 μL anti-strep magnetic beads (cat. no. 2-4090-002, IBA Lifesciences) for 30 min at room temperature. Unbound proteins were removed by washing three times with TBST buffer (25 mM Tris-HCl, pH 7.5, 150 mM NaCl, 0.05% Tween-20). Bound protein complex was eluted with LDS Sample Buffer (cat. no. NP0008, Invitrogen) containing 200 mM DTT and boiled at 95 °C for 10 min. Proteins were separated by SDS-PAGE (cat. no. M41215C, GenScript, Nanjing, China) and then transferred to a PVDF membrane. Immunoblotting analysis was conducted by incubation with corresponding primary antibodies and HPR-conjugated secondary antibodies. The band intensity of protein expression was quantified by Image J.

### Mass spectrometry analysis

*OLFML3* was overexpressed by transfection of H1-HeLa cells with OLFML3-myc-encoding plasmid. Empty vector was transfected as a negative control. Cells were lysed with IP lysis buffer, and the supernatant was collected by centrifugation at 13, 000 g for 5 min at 4 °C. OLFML3-interacting proteins were enriched from lysate by immunoprecipitation with anti-Myc magnetic beads and eluted in PBS. Samples were re-dissolved in 8 M urea solution for 1 h and then diluted with 100 mM ammonium bicarbonate buffer. Proteins were digested to peptides by trypsin treatment for 16 h at 37 °C. The digested peptides were desalted and loaded to Easy-nLC1200 UHPLC (Thermo Fisher Scientific) for mass spectrometry analysis. Data were acquired in the Orbitrap analyzer (Orbitrap Fusion, Thermo Fisher Scientific) and analyzed using Protein Discoverer 2.2 at the Analytical Chemistry Platform at SIAIS, ShanghaiTech University. The false discovery rate was set to 0.01. The proteins identified by mass spectrometry are listed in **Supplementary Table 7**.

### Immunofluorescence and confocal microscopy

H1-HeLa cells were seeded onto glass coverslips at low density and transfected with TRIM21-HA- and OLFML3-Flag-encoding plasmids. At 24 h after transfection, the cells were washed with ice-cold PBS three times, then fixed with 4% paraformaldehyde for 20 min at room temperature, and blocked with 3% BSA for 1 h at room temperature. The cells were incubated with anti-HA or anti-Flag antibody overnight at 4 °C, followed by staining with fluorescent dye-linked secondary antibodies for 1 h at room temperature. The nuclei were counterstained with Hoechst 33342 for 15 min at room temperature. The treated samples were visualized using confocal microscopy (LSM 710).

### Ubiquitination

HEK-293T cells were transfected with HA-tagged expression plasmids and Flag-tagged wild-type or mutant ubiquitin (**Supplementary Table 8**) in the absence or presence of His-tagged OLFML3 plasmids. At 24 h after transfection, the cells were treated with 10 mM MG132 for 6 h to inhibit protein degradation. Cells were collected and immunoprecipitated with anti-HA beads and further analyzed using immunoblotting with anti-Flag antibody for the ubiquitination test.

### Viral infection *in vivo*

Male mice on a C57BL/6 background at 8 to 9 weeks were used in this study. For the ELISA of serum IFN-β, *Olfml3*^+/+^ and *Olfml3*^-/-^ mice were intravenously injected with VSV at a dosage of 1.5 × 10^6^ PFU per gram body weight. At 24 after infection, blood was collected from the orbital sinus. For the other virus challenge experiments on mice, VSV was intraperitoneally injected at the same dosage, and tissues, including lungs, spleens, and livers, were harvested. For survival experiments, mice were monitored for 5 to 7 days after the VSV challenge.

### Surface plasmon resonance experiment

To determine the binding affinity of OLFML3 (MCE, cat. no. HY-P704162) for TRIM21 (MCE, cat. no. HY-P71791) and for an anti-OLFML3 antibody (Thermo, cat. no. PA531581), the SPR experiments were performed on a Biacore 8K (Cytiva) instrument. All experiments were conducted at 25°C using PBS supplemented with 0.05% Tween-20 and 0.1% BSA. OLFML3 was immobilized on flow cell 2 (FC2) of Series S Sensor Chip CM5 (Cytiva) using standard amine coupling chemistry. Briefly, the sensor was activated with a freshly mixed solution of 1-ethyl-3-(3-dimethylaminopropyl) carbodiimide (EDC) and N-hydroxysuccinimide (NHS) (Cytiva, Marlborough, MA, USA), followed by an injection of OLFML3 diluted in immobilization buffer (10 mM sodium acetate, pH optimized as needed). The remaining activated carboxyls were blocked using ethanolamine, and flow cell 1 (FC1) was left untreated and used as the reference surface. TRIM21 or anti−OLFML3 antibody (analytes) was prepared in running buffer and injected over FC1 and FC2 at 30 μL/min with an association phase of 180 s and a dissociation phase of 180 s. Binding responses were recorded using a twofold or 10-fold serial dilution series of analyte. After each cycle, the surface was regenerated using an appropriate regeneration solution to remove the bound analyte and restore the baseline. All binding data were double-referenced by blank cycle and reference flow cell subtraction. Binding curves were displayed, and the dissociation constants (*K*_D_) for the interaction were determined using the steady-state affinity method in Biacore 8 K Evaluation Software Version 3.0 (Cytiva).

### Statistical analysis

All data were collected from at least three independent biological replicates and presented as mean ± SD. Statistical analysis was calculated using two-tailed Student’s *t*-test by GraphPad Prism 8, unless noted otherwise. For mouse survival, Gehan–Breslow–Wilcoxon test was performed with GraphPad Prism 8 for analysis of statistical significance.

## Results

### OLFML3 antagonizes type I IFN signaling in RNA virus infection in mammalian cells

In our previous study, OLFML3 was shown to suppress type I IFN signaling in rhinovirus infection (21). We sought to investigate in the present study whether it is a general mechanism of OLFML3 to suppress innate immunity during RNA virus infection. We chose to conduct analyses on widespread influenza A virus A/PR8/34(H1N1) (PR8 IAV, NCBI: KC815527.1) and human coronaviruses 229E (NCBI: OK662398.1) and OC43 (NCBI: PQ630153.1) (**Supplementary Figure 1A**).

**Figure 1:**
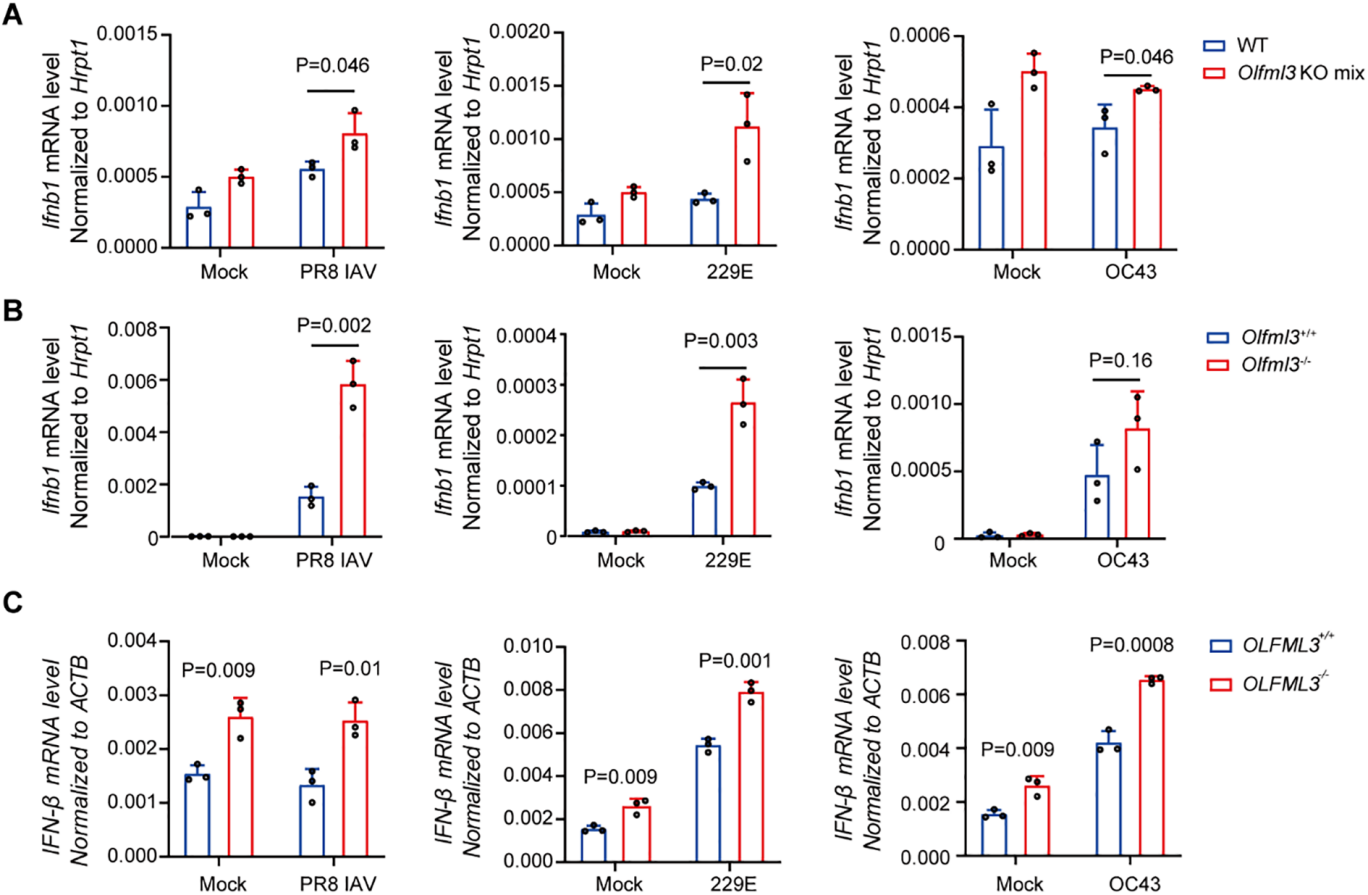
Evaluation of the function of OLFML3 in RNA virus infection *in vitro*. **(A–C)** RT-qPCR analysis of PR8 IAV and HCoV-229E- and HCoV-OC43-induced IFN-β mRNA expression in wild-type and *Olfml3* KO Mix MLE-12 mouse cells **(A)**, *Olfml3*^+/+^ and *Olfml3*^-/-^ mouse BMDMs **(B)**, and *OLFML3*^+/+^ and *OLFML3*^-/-^ A549 human cells **(C)**. *Olfml3*^-/-^, an isolated single clone of knockout cells. *Olfml3* KO Mix, mixed population of knockout cells. The cells are infected with viruses at an MOI of 1.0 for 24 h. The data are from three independent biological replicates and presented as mean ± SD. The significant difference is analyzed using two-tailed unpaired Student’s *t*-test. Source data are provided as a source data file.

It was found that *Olfml3* knockout in MLE-12 mouse lung epithelial cells (**Figure 1A**) and mouse bone marrow-derived macrophages (BMDMs) (22) (**Supplementary Figure 1B**; **Figure 1B**) enhanced the virus-induced mRNA expression of IFN-β. Consistently, *Olfml3* knockdown promoted IFN-β expression in MLE-12 mouse cells in response to RNA virus infection (**Supplementary Figure 1C**). Similarly, *OLFML3*^-/-^ A549 human lung carcinoma cells showed a higher IFN-β expression than wild-type A549 cells in the absence and presence of RNA viruses (**Figure 1C**). These data collectively demonstrated that OLFML3 served as a broad negative regulator of type I IFN production in RNA virus infection *in vitro*.

### OLFML3 negatively regulates vesicular-stomatitis-virus-induced IFN production *in vitro* and *in vivo*

**Figure 2:**
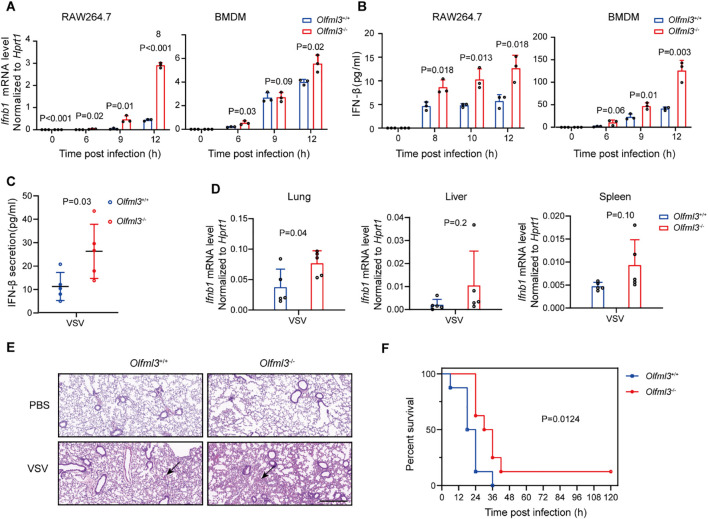
OLFML3 negatively regulates VSV-induced type I IFN production *in vitro* and *in vivo*. **(A)** RT-qPCR analysis of the effects of *Olfml3* knockout on VSV-induced *Ifnb1* mRNA expression in RAW264.7 cells and primary BMDMs. **(B)** ELISA analysis of secreted IFN-β protein in the supernatant of *Olfml3^+/+^* and *Olfml3^-/-^* RAW264.7 cell and BMDM culture in response to VSV infection. For **(A, B)**, cells were infected with VSV at a multiplicity of infection (MOI) of 0.3, and the samples were collected at the indicated time points. The data are from three independent biological replicates and presented as mean ± SD. The significant difference is analyzed using two-tailed unpaired Student’s *t*-test. **(C)** Enzyme-linked immunosorbent assay (ELISA) analysis of serum IFN-β protein in *Olfml3*^+/+^ and *Olfml3*^-/-^ C57BL/6 mice challenged with intravenously injected VSV (*n* = 5 per group) for 24 h. **(D)** RT-qPCR analysis of IFN-β mRNA expression in the lung, liver, and spleen of *Olfml3*^+/+^ and *Olfml3*^-/-^ mice (*n* = 5 per group) challenged with intraperitoneally injected VSV (*n* = 5 per group) for 24 h. For **(C, D)**, the significant difference is analyzed using two-tailed unpaired Student’s *t*-test. **(E)** Hematoxylin and eosin (H&E) staining of lung sections from *Olfml3*^+/+^ and *Olfml3*^-/-^ mice. Scale bar, 500 μm. **(F)** Survival curve of VSV-infected *Olfml3*^+/+^ and *Olfml3*^-/-^ mice (*n* = 8 per group). The significant difference is analyzed using Gehan–Breslow–Wilcoxon test. For *in vivo* experiments in **(E, F)**, VSV was administrated via an intraperitoneal injection at a dosage of 1.5 × 10^6^ PFU per gram body weight, and the samples were collected at 24 h post-infection or monitored over a course of 5 days. Source data are provided as a source data file.

To expand the scope of this study beyond respiratory viruses, we examined the function of OLFML3 in VSV (NCBI: OP734420.1) (**Supplementary Figure 2A**) infection *in vitro* and *in vivo*. We first analyzed the effects of *Olfml3* knockout on macrophages in response to VSV infection. It was found that during a time course of 12 h, *Olfml3^-/-^* RAW264.7 cells and mouse BMDMs consistently showed increased IFN-β expression as mRNA transcripts (**Figure 2A**) and as secreted protein (**Figure 2B**). In consistency with the results of IFN-β production, *Olfml3^-/-^* RAW264.7 cells and BMDMs showed significantly higher ISG expression (**Supplementary Figure 2B**) and lower viral loads (**Supplementary Figure 2C**) than the corresponding *Olfml3^+/+^* cells.

To understand the role of OLFML3 on type I IFN signaling *in vivo*, we first challenged wild-type (*Olfml3^+/+^*) and *Olfml3^-/-^* C57BL/6 mice with VSV via intravenous injection. A significantly higher level of VSV-induced serum IFN-β was observed in *Olfml3^-/-^* C57BL/6 mice (**Figure 2C**). The effects of *Olfml3* knockout on IFN-β mRNA expression in response to intraperitoneally injected VSV seemed to vary across tissues (**Figure 2D**). Nevertheless, *Olfml3* knockout resulted in consistently higher ISG expression across tissues (**Supplementary Figures 3A–C**). Accordingly, *Olfml3^-/-^* mice had significantly lower VSV loads in the lung, liver, and spleen than *Olfml3^+/+^* mice (**Supplementary Figure 3D**). Immunohistochemistry analysis revealed more inflammatory cell infiltration in lung tissues in *Olfml3^-/-^* mice than in *Olfml3^+/+^* mice (**Figure 2E**). Most importantly, *Olfml3* knockout increased the survival rate of VSV-infected mice (**Figure 2F**). Collectively, these data showed that OLFML3 could promote VSV infection by restricting virus-induced type I IFN production.

### OLFML3 regulates the VSV- and viral-RNA-mimic-induced IRF3 phosphorylation and nuclear localization

**Figure 3:**
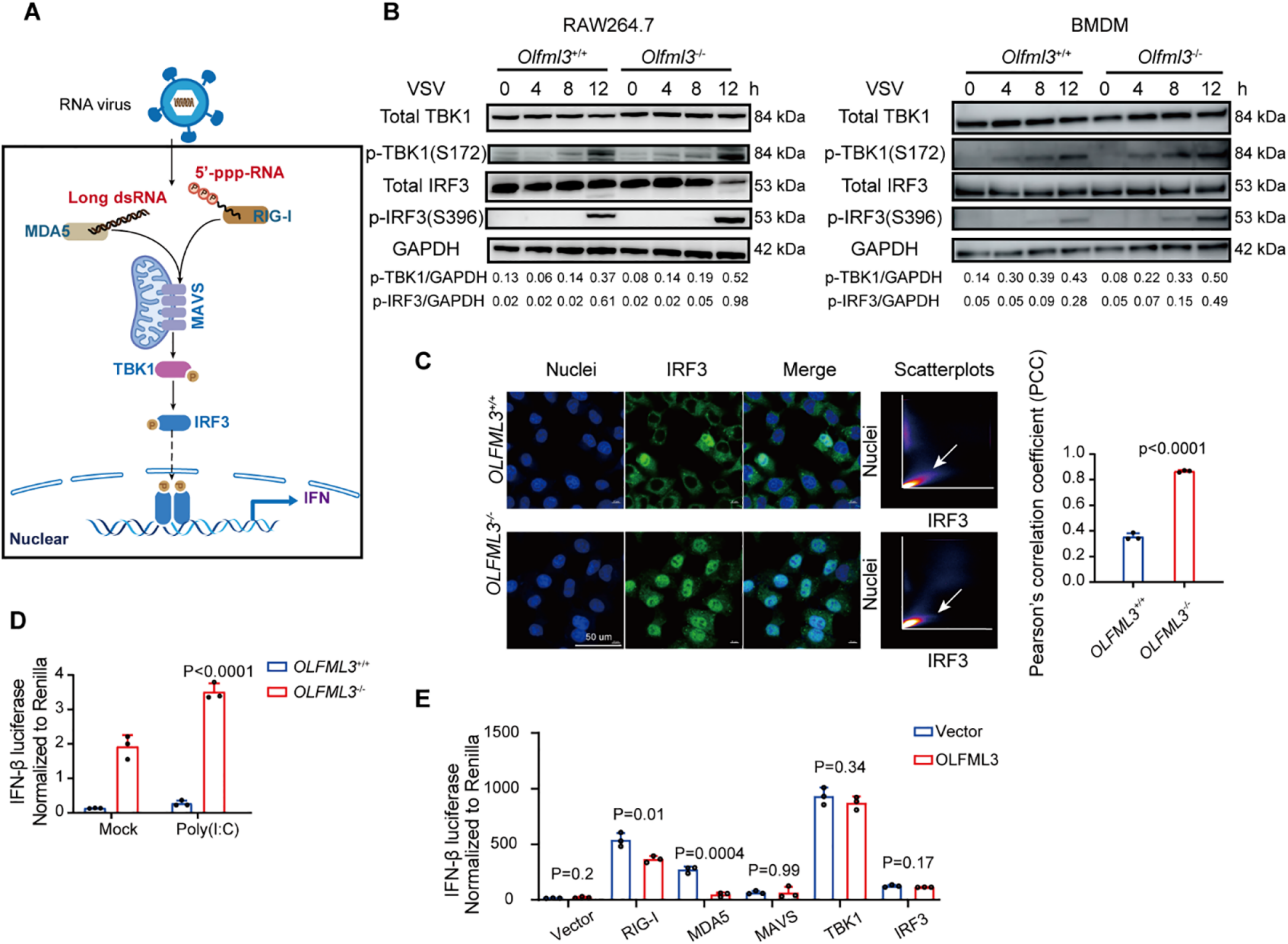
Evaluation of the effects of OLFML3 on VSV- and vRNA-induced IRF3 phosphorylation and nuclear translocation. **(A)** Cartoon showing RLR signaling in response to RNA virus infection. **(B)** WB analysis of phosphorylated TBK1 and IRF3 in VSV-treated *Olfml3^+/+^* and *Olfml3^-/-^* RAW264.7 cells and BMDMs. **(C)** Immunofluorescence microscopy analysis of nuclear localization of IRF3 in *OLFML3^+/+^* and *OLFML3^-/-^* H1-HeLa cells. The cells are treated with Poly(I:C) for 6 h. Scale bar, 50 μm. Nucleus-localized IRF3 is calculated by the co-localization of green (IRF3) and blue (nuclei) signals and shown as Pearson coefficient. **(D)** Effects of *OLFML3* knockout on Poly(I:C)-induced IFN-β promoter activation in HEK-293T cells, as determined by luciferase reporter assay. **(E)** Effects of *OLFML3* overexpression on node protein overexpression-induced IFN-β promoter activation in HEK-293T cells. For **(D, E)**, the data are from three independent biological replicates and presented as mean ± SD. The significant difference is analyzed using two-tailed unpaired Student’s *t*-test. The mock groups received PBS treatment without stimulation. Source data are provided as a source data file.

During the innate immune response to RNA virus infection, viral RNAs can be recognized by retinoic-acid-inducible gene I (RIG-I)-like receptors (RLRs), including RIG-I and melanoma differentiation-associated protein 5 (MDA5). Downstream RLR signaling involves the phosphorylation of TANK-binding kinase 1 (TBK1) and interferon regulatory factor 3 (IRF3). Phosphorylated IRF3 enters the nucleus, dimerizes, and then binds to the promoter to initiate type I IFN production (24, 25) (**Figure 3A**). In the present study, it was found that *Olfml3* knockout could enhance VSV-induced S172 phosphorylation of TBK1 and S396 phosphorylation of IRF3 in RAW264.7 cells and BMDMs (**Figure 3B**). These results consistently suggested an important role of OLFML3 in VSV-induced RLR signaling.

Next, we generated *OLFML3* knockout single clones of H1-HeLa cells and HEK-293T (**Supplementary Figure 4A**) and examined the effects of OLFML3 on IRF3 activation in response to the stimulation of Poly(I:C) and 5′-ppp RNA as viral RNA (vRNA) mimics. Consistent with the VSV infection in mouse macrophages, vRNA stimulation resulted in a significantly higher level of IFN-β (**Supplementary Figures 4B, C**) and ISG (**Supplementary Figure 4D**) production in *OLFML3*^-/-^ H1-HeLa cells than in *OLFML3*^+/+^ cells. Importantly, Poly(I:C) and 5′-ppp RNA stimulation resulted in a higher level of TBK1 phosphorylation (**Supplementary Figure 4E**) and IRF3 phosphorylation (**Supplementary Figure 4F**). Moreover, immunofluorescence experiments showed that *OLFML3*^-/-^ H1-HeLa cells had increased nuclear translocation of IRF3 in response to Poly(I:C) stimulation compared with *OLFML3*^+/+^ cells (**Figure 3C**).

We then investigated the acting point of OLFML3 along the RLR signaling. We constructed a luciferase reporter as described (26, 27), where the expression of firefly luciferase was under the control of IFN-β promoter and the overexpression of RLR signaling proteins was used as stimuli for promoter activation. It was found that *OLFML3* knockout upregulated IFN-β promoter activation in the presence of Poly(I:C) stimulation (**Figure 3D**). More importantly, in the context of overexpression of RLR transducer proteins RIG-I, MDA5, mitochondrial antiviral signaling protein (MAVS), TBK1, and IRF3, *OLFML3* overexpression resulted in a difference in IFN-β promoter activation only in the case of RIG-I and MDA5 overexpression (**Figure 3E**). These results suggested that OLFML3 regulated IFN production through canonical IRF3-mediated RLR signaling, with a possible role at the upstream vRNA sensor proteins RIG-I and MDA5.

### OLFML3 destabilizes RIG-I and interacts with E3 ubiquitin ligase tripartite motif protein 21

**Figure 4:**
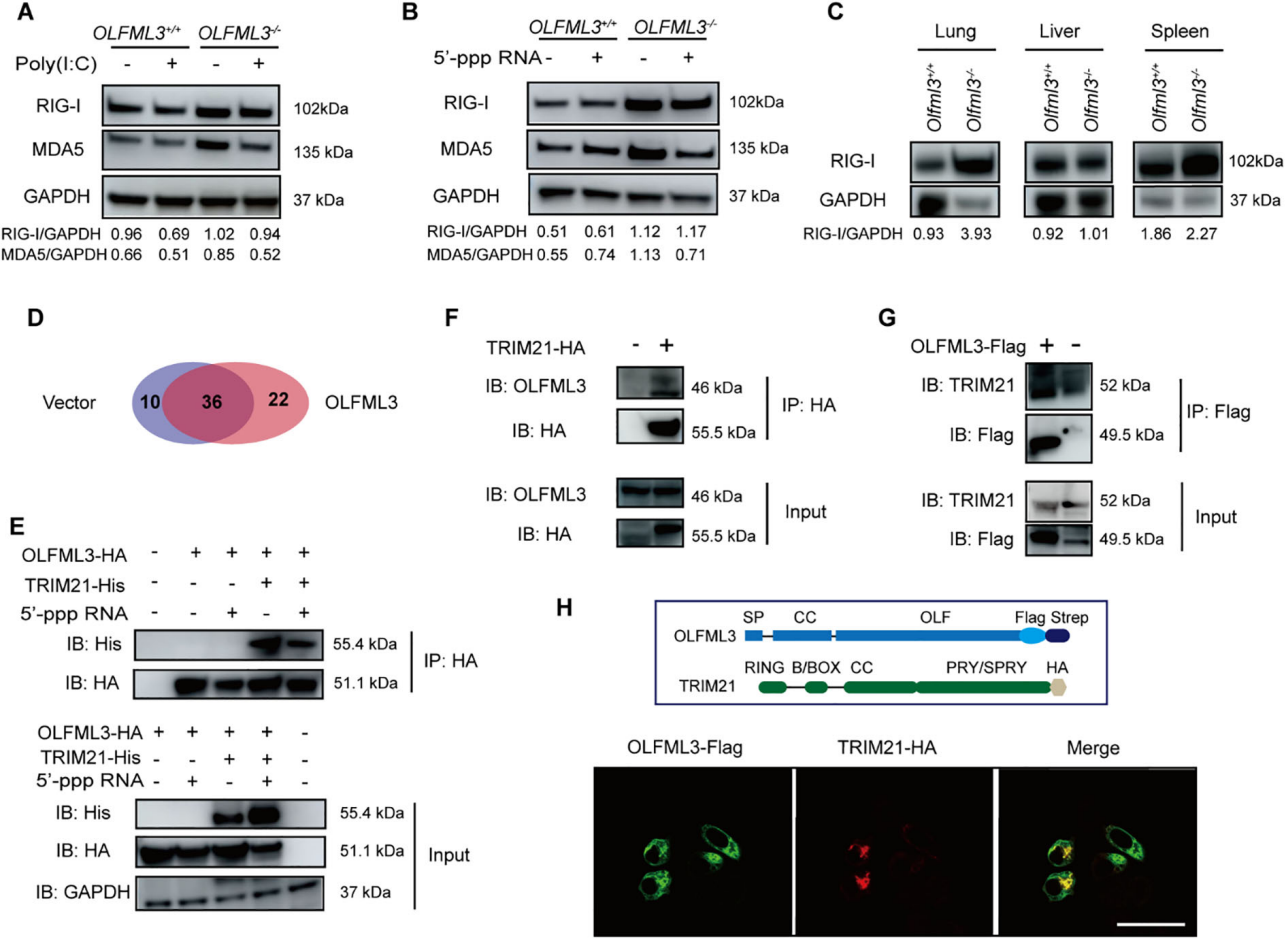
OLFML3 destabilizes RIG-I and interacts with TRIM21. **(A, B)** WB analysis of the effects of *OLFML3* knockout on RLRs in H1-HeLa cells. The cells are treated with mock transfection, Poly(I:C) **(A)**, or 5′-ppp RNA **(B)**, and the samples were collected at 6 h after transfection. The data for additional replicates can be found in **Supplementary Figures 4A, B**. **(C)** WB analysis of RIG-I expression in the tissues of *Olfml3*^+/+^ and *Olfml3*^-/-^ mice. **(D)** Venn plot showing OLFML3-interacting proteins identified through unbiased MS analysis. H1-HeLa cells are transfected with Myc-tagged OLFML3 (OLFML3-Myc)-encoding or empty plasmids and then immunoprecipitated with Myc-binding beads. A *q*-value cutoff less than 0.005 is applied. **(E)** Co-IP analysis of the interaction between OLFML3-HA and TRIM21-His in HEK-293T cells. **(F, G)** Co-IP analysis of the interactions between TRIM21-HA and endogenous OLFML3 **(F)** and between OLFML3-Flag and endogenous TRIM21 **(G)** in RAW264.7 cells. **(H)** Immunofluorescence microscopy analysis of the co-localization of TRIM21-HA and OLFML3-Flag in H1-HeLa cells. Scale bar, 50 μm. For TRIM21, the domains are RING for Really Interesting New Gene domain, B/BOX for B/BOX domain, CC for coiled-coil domain, and PRY/SPRY for PRY and SPRY domain. For OLFML3, the domains are SP for signal peptide, CC for coiled-coil domain, and OLF for olfactomedin domain. Source data are provided as a source data file.

In order to validate the action of OLFML3 on vRNA sensor proteins, we analyzed the cellular protein levels of RIG-I and MDA5 in the absence and presence of OLFML3. It was found that *OLFML3* knockout increased the RIG-I protein levels in the presence of Poly(I:C) and 5′-ppp RNA (**Figures 4A, B**), suggesting a role of OLFML3 in destabilizing RIG-I. These results were consistently observed in independent biological replicates (**Supplementary Figures 5A, B**). Gain-of-function analysis consistently showed that *OLFML3* overexpression in *OLFML3*^-/-^ H1-HeLa cells reduced the protein levels of RIG-I (**Supplementary Figure 5C**). We also noted that *Olfml3^-/-^* mice exhibited a higher RIG-I protein level at several tissues examined, such as lung and spleen, than *Olfml3^+/+^* mice (**Figure 4C**). Collectively, these results illustrated that OLFML3 could destabilize RIG-I, which might, in turn, lead to reduced type I IFN production.

To the best of our knowledge, OLFML3 has not been reported to directly participate in protein stability. Thus, we performed an unbiased mass spectrometry (MS) analysis to identify the cellular co-factors of OLFML3 that may be involved in RIG-I stability. By excluding non-specific hits in empty plasmid control, 22 OLFML3-specific hits were identified in H1-HeLa cells (**Figure 4D**). Among these hits, E3 ubiquitin ligase tripartite motif protein 21 (TRIM21) came to our attention. TRIM21 is known to be involved in multiple processes of innate immunity (28–34), but its cellular function with RLRs has not been reported so far.

We then sought to validate the cellular interaction between OLFML3 and TRIM21 across different cell types using multiple techniques. It was found that overexpressed HA-tagged OLFML3 (OLFML3-HA) and His-tagged TRIM21 (TRIM21-His) were co-immunoprecipitated (Co-IP) in HEK-293T cells (**Figure 4E**). In RAW264.7 cells, the interactions between TRIM21-HA and endogenous OLFML3 (**Figure 4F**) and between OLFML3-Flag and endogenous TRIM21 (**Figure 4G**) were also validated by Co-IP analysis. Moreover, immunofluorescence microscopy analysis revealed the co-localization of OLFML3-Flag and TRIM21-HA in H1-Hela cells (**Figure 4H**). As OLFML3 has an N-terminal peptide, we intended to investigate whether the cytoplasmic form of OLFML3 was responsible for the observed immunosuppressive function. We generated a secretion-deficient mutant by deleting its signal peptide (**Δ**SP-Olfml3) and found that **Δ**SP-Olfml3 suppressed VSV-induced type I IFN production with similar efficiency to the full-length Olfml3 (**Supplementary Figure 5D**).

### TRIM21 is involved in RIG-I-mediated type I IFN production and is necessary for the immunosuppressive function of OLFML3

**Figure 5:**
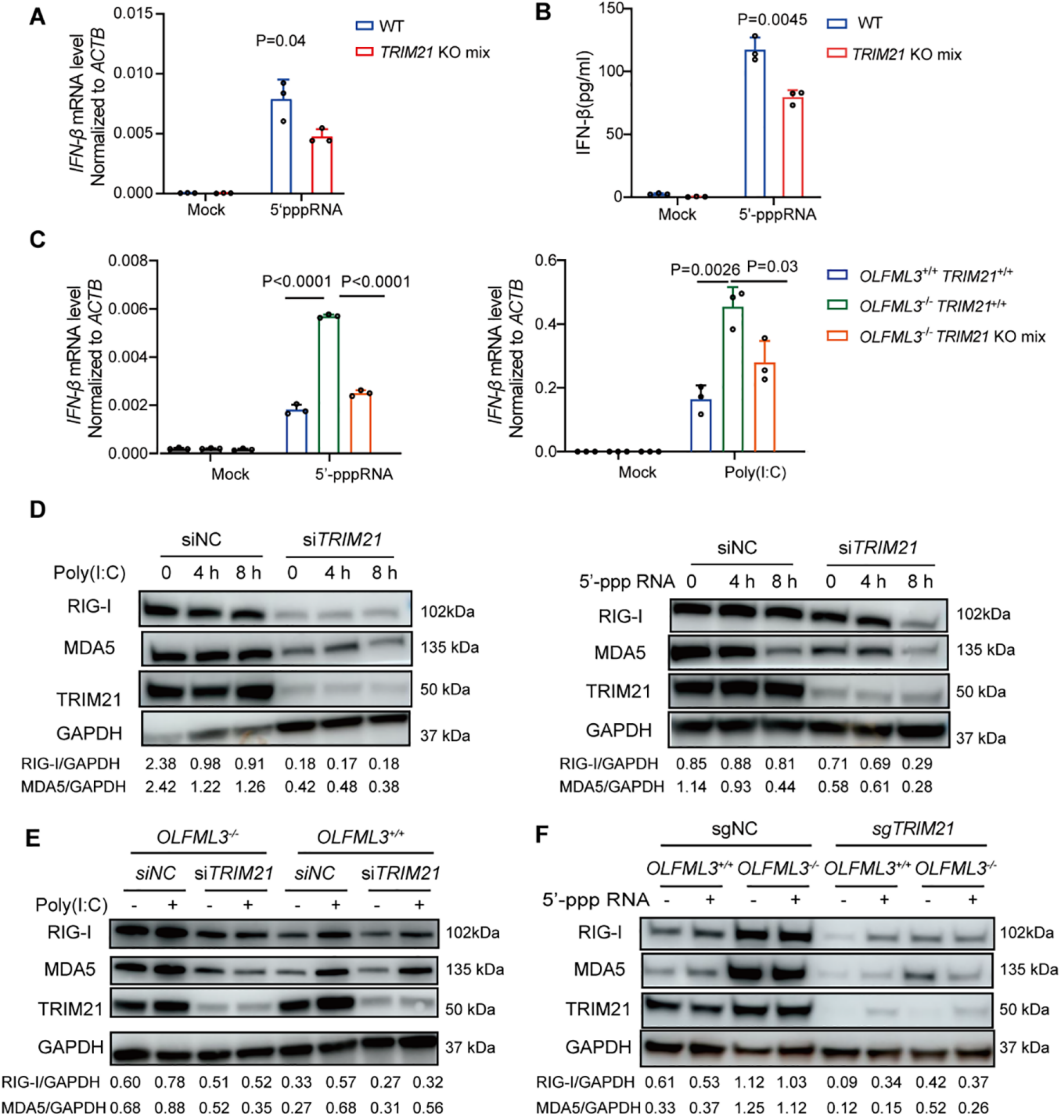
Effects of TRIM21 on vRNA-induced type I IFN production and RLR stability. **(A, B)** RT-qPCR and ELISA analysis of the effects of *TRIM21* knockout on IFN-β mRNA **(A)** and secreted protein **(B)**, respectively, in H1-HeLa cells. The cells are transfected with 5′-pppRNA, and the samples were collected at 9 h post-transfection. KO mix, mixed population of knockout cells without isolation of single clones. **(C)** RT-qPCR analysis of the effect of *TRIM21* knockout on vRNA-induced IFN-β mRNA expression in *OLFML3^+/+^* and *OLFML3^-/-^* H1-HeLa cells. The cells are treated with Poly(I:C) or 5′-pppRNA for 9 h. **(D)** WB analysis of the effects of *TRIM21* knockdown by siRNA (si*TRIM21*) on RIG-I and MDA5 protein levels. H1-HeLa cells are treated with Poly(I:C) or 5′-pppRNA for the indicated times. **(E)** WB analysis of the effects of dual *TRIM21/OLFML3* depletion on RLR protein level. *OLFML3*^+/+^ and *OLFML3*^-/-^ H1-HeLa cells are treated with mock or Poly(I:C) for 9 h. **(F)** WB analysis of the effects of dual *TRIM21/OLFML3* depletion on RLR protein levels. *OLFML3*^+/+^ and *OLFML3*^-/-^ H1-HeLa cells are treated with 5′-pppRNA for 9 h. For **(E, F)**, NC means negative control with scrambled siRNA or sgRNA. sgTRIM21 refers to a mixed population of *TRIM21* knockout cells without isolation of single clones. Source data are provided as a source data file.

With confirmed interaction between TRIM21 and OLFML3, we next asked whether and how TRIM21 was involved in vRNA-induced type I IFN production. It was found that *TRIM21* knockout in H1-HeLa significantly reduced 5′-pppRNA-induced IFN-β mRNA (**Figure 5A**) and protein (**Figure 5B**) production. We then constructed dual knockout cells to investigate whether OLFML3 and TRIM21 played additive or opposite roles in type I IFN production. It was found that *OLFML3* knockout increased vRNA-induced IFN-β mRNA expression and secreted protein, while *TRIM21*/*OLFML3* dual knockout reduced IFN-β production to nearly wild-type levels (**Figure 5C**; **Supplementary Figure 5E**). These data illustrated that TRIM21 promoted vRNA-induced IFN-β production, which was against the function of OLFML3.

As the results above showed that OLFML3 destabilized RLR, we asked whether TRIM21 played an opposite role against OLFML3 in RLR stability. The WB analysis showed that *TRIM21* knockout reduced the protein level of RIG-I (**Supplementary Figure 5F**). *TRIM21* knockdown using small interfering RNA (siRNA) consistently revealed that downregulated *TRIM21* expression resulted in decreased RIG-I and MDA5 protein levels in the absence and presence of Poly(I:C) and 5′-pppRNA (**Figure 5D**). These data elucidated that TRIM21 stabilized the cellular RIG-I levels, which was against the function of OLFML3 in RIG-I stability.

Then, we sought to explore whether OLFML3 and TRIM21 functioned in the same pathway and whether their interaction, as revealed by the Co-IP results above, had physiological relevance with regard to RLR stability. Dual loss-of-function analysis was thus performed by simultaneously depleting cellular OLFML3 and TRIM21. OLFML3 depletion was achieved using *OLFML3^-/-^* single clone of H1-HeLa cells, while TRIM21 depletion was achieved using either siRNA (**Figure 5E**) or CRISPR-based knockout (**Figure 5F**). It was found that in the presence of TRIM21 (sgNC), OLFML3 depletion significantly increased 5′-ppp RNA-induced RIG-I expression (**Figure 5F**; 1.03 versus 0.53 by comparing lane 2 and lane 4), whereas *TRIM21* knockout abolished the effects of OLFML3 on 5′-ppp RNA-induced RIG-I expression (**Figure 5F**; 0.34 versus 0.37 by comparing lane 6 and lane 8). These results suggested that the action of OLFML3 on stimulus-induced RIG-I expression was dependent on TRIM21.

### OLFML3-OLF domain interacts with TRIM21-PRY/SPRY domain

**Figure 6:**
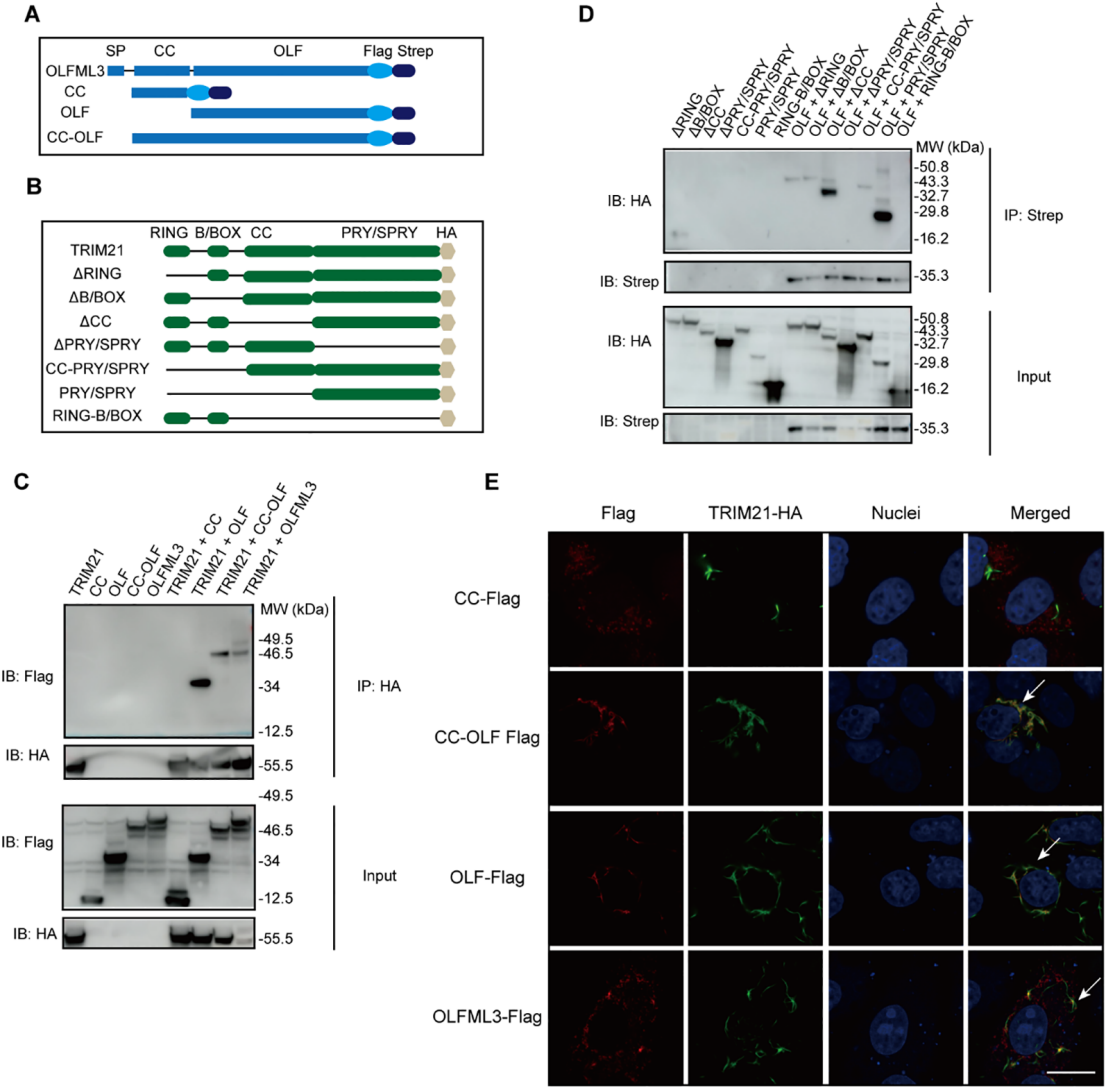
Evaluation of the pattern of interactions between OLFML3 and TRIM21. **(A)** Design of OLFML3 constructs with Flag-Strep tags. **(B)** Design of TRIM21 constructs with HA tags. **(C)** Co-IP analysis of the interactions between TRIM21 and individual domains of OLFML3. **(D)** Co-IP analysis of the interactions between OLF domain of OLFML3 and individual domains or truncation constructs of TRIM21. **(E)** Immunofluorescence analysis of the co-localization of TRIM21 and OLFML3 domains in H1-HeLa cells. The cells are transfected with corresponding plasmids for 24 h and then stained with antibodies. Scale bar, 25 μm. Source data are provided as a source data file.

Since the results above showed that the interaction between OLFML3 and TRIM21 was important for type I IFN production and RIG-I stability, we intended to dissect the molecular mechanism of the interaction. OLFML3 is composed of signal peptide (SP), coiled-coil (CC), and olfactomedin (OLF) domains (7) (**Figure 6A**). TRIM21 consists of RING, B/BOX, CC, and PRY/SPRY domains (32) (**Figure 6B**). We designed several molecular constructs encoding individual domains and truncation mutants of OLFML3 and TRIM21 (**Figures 6A, B**) to determine the interacting modules on each protein. It was found that TRIM21 could be co-immunoprecipitated with both OLF, CC-OLF, and full-length OLFML3, but not with CC domain alone (**Figure 6C**), suggesting that OLF was the essential module for OLFML3 to interact with TRIM21. We then used OLF domain of OLFML3 as a bait to determine the domains in TRIM21 that could interact with OLFML3. It was found that OLF domain could be co-immunoprecipitated with ΔRING, ΔB/BOX, ΔCC, CC-PRY/SPRY, and PRY/SPRY constructs, but not with ΔPRY/SPRY or RING-B/BOX construct (**Figure 6D**). These results suggested that the PRY/SPRY domain from TRIM21 was most likely responsible for the interaction with OLF. In addition, immunofluorescence microscopy analysis revealed the co-localization of OLF domain and TRIM21 in H1-Hela cells (**Figure 6E**). Collectively, these results revealed that the interaction between OLFML3 and TRIM21 occurred between OLF and PRY/SPRY domains under cellular conditions.

Surface plasmon resonance (SPR) assays showed that while mobilized OLFML3 had strong binding with anti-OLFML3 antibody (**Supplementary Figures 6A, B**), it did not show apparent binding with TRIM21 protein (**Supplementary Figures 6C, D**). These results indicated that OLFML3 likely modulated TRIM21 function through an indirect mechanism rather than direct interaction.

### OLFML3 inhibits TRIM21-mediated K63 ubiquitination of RIG-I

**Figure 7:**
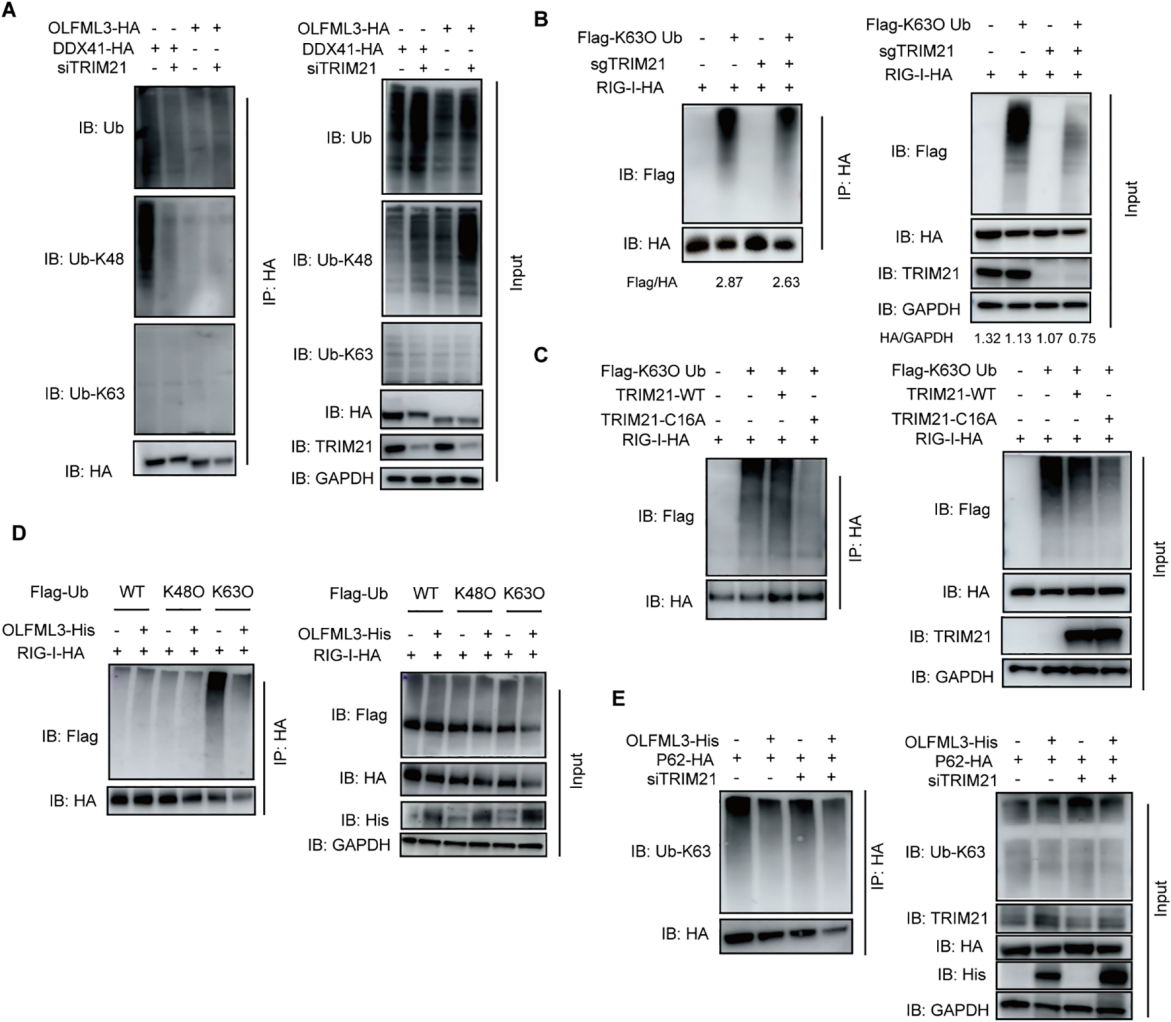
Mechanism of action of OLFML3 on inhibiting TRIM21 activity. **(A)** Co-IP analysis of the effects of *TRIM21* knockdown on DDX41 and OLFML3 ubiquitination in HEK-293T cells. **(B)** Co-IP analysis of the effects of *TRIM21* knockout on K63 ubiquitination of RIG-I in HEK-293T cells. **(C)** Co-IP analysis of the effects of TRIM21 wide type or C16A mutant on K63 ubiquitination of RIG-I in HEK-293T cells (mixed population). **(D)** Co-IP analysis of the effects of *OLFML3* overexpression on RIG-I ubiquitination in HEK-293T cells. For **(B, D)**, K48O Ub, ubiquitin with the only lysine residue at position 48; K63O Ub, ubiquitin with the only lysine residue at position 63. **(E)** Co-IP analysis of the effects of OLFML3 overexpression and TRIM21 knockdown on K63 ubiquitination of P62 protein in HEK-293T cells.

Because PRY/SPRY is the substrate binding domain of the E3 ubiquitin ligase TRIM21, we investigated whether OLFML3 could be ubiquitinated by TRIM21 as a substrate protein. It has been reported that TRIM21 could mediate Lys48 (K48)-linked ubiquitination and the subsequent proteasomal degradation of DDX41, IRF7, and IRF3 (35–38). Co-IP analysis showed that *TRIM21* knockdown significantly reduced the K48 ubiquitination of DDX41 but had little effects on the total, K48, and K63 ubiquitination of OLFML3 (**Figure 7A**). These suggested that OLFML3 was not a substrate protein of TRIM21, though it bound to the PRY/SPRY substrate-binding domain of TRIM21.

It has been reported that TRIM21 can stabilize substrate proteins by mediating K63 ubiquitination, which competes K48 ubiquitination and thereby prevents the proteasomal degradation of substrate proteins (39). Thus, we speculated that TRIM21 could stabilize RIG-I by mediating the K63 ubiquitination of RIG-I. Co-IP analysis showed that *TRIM21* knockout reduced cellular RIG-I (**Figure 7B**; 0.75 versus 1.13 in lane 4 and lane 2), consistent with the results of TRIM21 knockdown (**Figures 5D–F**). In addition, K63-ubiquitinated RIG-I was modestly reduced in *TRIM21* knockout cells. These results indicated that TRIM21 mediated RIG-I stabilization and its K63 ubiquitination (**Figure 7B**). Furthermore, we constructed a dead mutant of TRIM21, TRIM21 (C16A), that was deficient of E3 ligase activity (**Supplementary Figure 7A**). Co-IP analysis revealed that TRIM21 (C16A) inactive mutant displayed reduced activity on K63-linked ubiquitination of RIG-I (**Figure 7C**). To the best of our knowledge, this was the first report for TRIM21-mediated K63 ubiquitination of RIG-I.

Importantly, *OLFML3* overexpression significantly inhibited K63 ubiquitination of RIG-I with little effects on its K48 ubiquitination (**Figure 7D**). Interestingly, *OLFML3* knockout appeared to enhance the K48 ubiquitination of DDX41, IRF7, and IRF3 (**Supplementary Figures 7B–D**), suggesting that the effects of OLFML3 on the K48 ubiquitination activity of TRIM21 might be substrate dependent. To explore whether the effects of OLFML3 on the K63 ubiquitination activity of TRIM21 is limited to RIG-I, we investigated the K63-linked ubiquitination of P62, which is a known substrate of TRIM21 (40–43). It was found that *OLFML3* overexpression and *TRIM21* knockdown could both inhibit the K63 ubiquitination of P62 protein, with possible additive effects (**Figure 7E**). These results suggested that the effects of OLFML3 on the K63 ubiquitination activity of TRIM21 was not limited to RIG-I. Collectively, our data suggested that OLFML3 destabilized RIG-I by inhibiting the TRIM21-mediated K63 ubiquitination of RIG-I. Similarly, OLFML3 could inhibit the K63 ubiquitination of MDA5 (**Supplementary Figure 7E**).

## Discussion

Regulation of type I IFN signaling-mediated antiviral response is an essential component for maintaining immune homeostasis. Several negative regulators that are involved in RLR signaling-mediated type I IFN production have been identified (24, 25, 44–46). OLFML3 belongs to OLF protein family and is generally considered to be associated with tumorigenesis and development. Our previous studies have revealed the regulatory functions of OLFML3 in rhinovirus and bacterial infections. However, the breath and molecular mechanisms of OLFML3 in virus infections remain unknown.

OLFM4, a close OLF family member of OLFML3, has been widely reported to play a modulatory role in bacterial infection and inflammation response (47–49). These results prompted us to investigate a more broad-spectrum function of OLFML3 in virus infection. In the present study, we found that OLFML3 negatively regulated type I IFN production across diverse categories of RNA viruses, including influenza viruses, coronaviruses, and VSV (**Figures 1**, **2**). This modulatory effect was achieved by destabilizing RIG-I in a TRIM21-dependent manner (**Figures 3**-**5**). To the best of our knowledge, the present study is the first report on TRIM21-mediated K63 ubiquitination of RIG-I. This result was further supported by mutational analyses with TRIM21-C16A dead mutant (**Figure 7**).

One interesting discovery was that while OLFML3 bound to the PRY/SPRY domain of TRIM21 (**Figure 6**) and inhibited its K63 ubiquitination activity on RIG-I as well as other substrate proteins, OLFML3 itself was not a substrate of TRIM21. Another interesting discovery was that the inhibitory effects of OLFML3 on TRIM21 seemed to be applicable to both K48 and K63 ubiquitination activity (**Figure 7**; **Supplementary Figure 7**). Moreover, the inhibitory effects of OLFML3 on TRIM21-RIG-I signaling axis were independent of viral or RNA stimulation (**Figures 4**, **5**). These results suggest that OLFML3 may function as a consistently active “off-switch” and participate in maintaining the homeostasis of host innate immunity. It would be thus interesting to investigate in future studies the immunomodulatory function of OLFML3 in a broader context in addition to viral infection.

The Co-IP and SPR experiments collectively suggested that OLFML3 interacted with TRIM21 via an indirect mechanism under physiological conditions. OLFML3 could act as a scaffold (7) or adaptor, recruiting a yet unidentified regulatory protein that specifically interacts with TRIM21 to form a functional ternary complex. We hypothesize that this complex could specifically inhibit the K63-linked ubiquitination of RIG-I by TRIM21, thereby attenuating the downstream type I interferon (IFN-I) signaling cascade and exerting an immunosuppressive effect.

Our study revealed the OLFML3–TRIM21–RIG-I axis as a universal mechanism for OLFML3-mediated suppression of type I IFN signaling in RNA virus infection. However, the molecular basis for inhibition of TRIM21 activity remains unclear. It is known that TRIM21 can dimerize or oligomerize via its coiled-coil domain. Given the observation that TRIM21 activity was likely inhibited in an allosteric manner, it is thus possible that an unidentified regulatory protein disturbs TRIM21 dimerization or oligomerization. Another limitation of the present study was that Co-IP-MS identified a list of binding proteins of OLFML3 (**Figure 4**), and many of these have not been investigated. The present study only focused on partner proteins that could have effects on the cellular protein levels of RIG-I. Therefore, there could be other mechanisms for OLFML3 to exert a broad-spectrum inhibitory activity on type I IFN production. In addition, this study was oriented to analyze the effects of OLFML3 on RIG-I at the translational level, whereas the effects of OLFML3 on RNA virus infections have not been examined at the epigenetic, transcriptional, or post-translational levels.

Importantly, it has to be noted that the present study focused on the functions of OLFML3 on RNA virus infection, and whether OLFML3 has a similar activity in DNA virus infection remains to be explored. Specifically, some DNA viruses such as human adenoviruses (HAdVs) can produce viral-associated RNAs (50–52), which also triggers RLR signaling in host cells. Therefore, it would be interesting to examine whether OLFML3 can inhibit RLR-mediated type I IFN production in DNA virus infections and, if so, whether the same mechanisms of action apply.

Although our results demonstrated that Olfml3 deficiency significantly enhanced type I IFN expression both *in vitro* and *in vivo*, the survival phenotype in VSV challenge model was relatively limited. This could be due to several different factors. First of all, Olfml3 is known to be a secreted protein. Its *in vivo* function as a secreted protein may interfere with its intracellular immunosuppressive function. Second, the *Olfml3*^-/-^ mice used in this study are full-body knockout that may complicate the analysis of the immunosuppressive function of Olfml3. Future investigation with tissue-specific or conditional knockout mice can help solve this issue. Finally, VSV challenge experiment is a relatively severe disease model. At the conditions of this study, most animals reached the endpoint within 48 h post-infection. Further virus challenge analyses with different levels of severity may better delineate the *in vivo* function of Olfml3.

In summary, our research identified that OLFML3 functioned as a negative regulator of RIG-I-mediated type I IFN production in RNA virus infection via the OLFML3–TRIM21–RIG-I axis. This study revealed a novel regulatory mechanism of RNA virus-induced innate immunity. These findings can help in understanding the homeostasis of immune system during viral infections. More importantly, our study may pave the way for the development of antiviral therapeutics targeting the OLFML3 axis, the suppression of which can re-activate type I IFN signaling.

## Data Availability

The original contributions presented in the study are included in the article, **Supplementary Material** and source data. Further inquiries can be directed to the corresponding authors.

## References

[1] Zeng LC Liu F Zhang X Zhu ZD Wang ZQ Han ZG . hOLF44, a secreted glycoprotein with distinct expression pattern, belongs to an uncharacterized olfactomedin-like subfamily newly identified by phylogenetic analysis. FEBS Lett. (2004) 571:74–80. doi: 10.1016/j.febslet.2004.06.059 15280020

[2] Anholt RRH . Olfactomedin proteins: central players in development and disease. Front Cell Dev Biol. (2014) 2:6. doi: 10.3389/fcell.2014.00006 25364714 PMC4206993

[3] Jin Y Li J-L . Olfactomedin-like 3: possible functions in embryonic development and tumorigenesis. Chin Med J (Engl). (2019) 132:1733–8. doi: 10.1097/cm9.0000000000000309 31261202 PMC6759097

[4] Zhao S Zhang J Hou X Zan L Wang N Tang Z . OLFML3 expression is decreased during prenatal muscle development and regulated by microRNA-155 in pigs. Int J Biol Sci. (2012) 8:459–69. doi: 10.7150/ijbs.3821 22419891 PMC3303172

[5] Chen H Li R Bian J Li X Su C Wang Y . OLFML3 suppresses trophoblast apoptosis via the PI3K/AKT pathway: A possible therapeutic target in preeclampsia. Placenta. (2024) 147:1–11. doi: 10.1016/j.placenta.2024.01.008 38277999

[6] Stalin J Imhof BA Coquoz O Jeitziner R Hammel P McKee TA . Targeting OLFML3 in colorectal cancer suppresses tumor growth and angiogenesis, and increases the efficacy of anti-PD1 based immunotherapy. Cancers (Basel). (2021) 13:4625. doi: 10.3390/cancers13184625 34572851 PMC8464773

[7] Inomata H Haraguchi T Sasai Y . Robust stability of the embryonic axial pattern requires a secreted scaffold for chordin degradation. Cell. (2008) 134:854–65. doi: 10.1016/j.cell.2008.07.008 18775317

[8] Miljkovic-Licina M Hammel P Garrido-Urbani S Lee BP Meguenani M Chaabane C . Targeting olfactomedin-like 3 inhibits tumor growth by impairing angiogenesis and pericyte coverage. Mol Cancer Ther. (2012) 11:2588–99. doi: 10.1158/1535-7163.mct-12-0245 23002094

[9] Imhof BA Ballet R Hammel P Jemelin S Garrido-Urbani S Ikeya M . Olfactomedin-like 3 promotes PDGF-dependent pericyte proliferation and migration during embryonic blood vessel formation. FASEB J. (2020) 34:15559–76. doi: 10.1096/fj.202000751rr 32997357

[10] Joseph LM Toedebusch RG Debebe E Bastian AH Lucchesi CA Syed-Quadri S . Microglia-derived olfactomedin-like 3 is a potent angiogenic factor in primary mouse brain endothelial cells: A novel target for glioblastoma. Int J Mol Sci. (2022) 23:4163. doi: 10.3390/ijms232314613 36498941 PMC9741462

[11] Dunn LL De Valence S Tille JC Hammel P Walpoth BH Stocker R . Biodegradable and plasma-treated electrospun scaffolds coated with recombinant olfactomedin-like 3 for accelerating wound healing and tissue regeneration. Wound Repair Regener. (2016) 24:1030–5. doi: 10.1111/wrr.12485 27684720

[12] Yadav H Bakshi A Anamika Singh V Paul P Murugan NA . Co-localization and co-expression of Olfml3 with Iba1 in brain of mice. J Neuroimmunol. (2024) 394:578411. doi: 10.1016/j.jneuroim.2024.578411 39079458

[13] Neidert N Von Ehr A Zoller T Spittau B . Microglia-specific expression of Olfml3 is directly regulated by transforming growth factor beta1-induced smad2 signaling. Front Immunol. (2018) 9:1728. doi: 10.3389/fimmu.2018.01728 30093905 PMC6070609

[14] Chen P Hsu W-H Chang A Tan Z Lan Z Zhou A . Circadian regulator CLOCK recruits immune-suppressive microglia into the GBM tumor microenvironment. Cancer Discov. (2020) 10:371–81. doi: 10.1158/2159-8290.cd-19-0400 31919052 PMC7058515

[15] Toedebusch RG Lucchesi CA Debebe ET Wittenburg LA Chen X Toedebusch CM . Microglia-derived olfactomedin-like 3 promotes pro-tumorigenic microglial function and Malignant features of glioma cells. Int J Mol Sci. (2021) 22:13052. doi: 10.3390/ijms222313052 34884869 PMC8657851

[16] Xuan W Hsu W-H Khan F Dunterman M Pang L Wainwright DA . Circadian regulator CLOCK drives immunosuppression in glioblastoma. Cancer Immunol Res. (2022) 10:770–84. doi: 10.1158/2326-6066.cir-21-0559 35413115 PMC9177794

[17] Pang L Dunterman M Xuan W Gonzalez A Lin Y Hsu WH . Circadian regulator CLOCK promotes tumor angiogenesis in glioblastoma. Cell Rep. (2023) 42:112127. doi: 10.1016/j.celrep.2023.112127 36795563 PMC10423747

[18] Liu Y Wu J Najem H Lin Y Pang L Khan F . Dual targeting macrophages and microglia is a therapeutic vulnerability in models of PTEN-deficient glioblastoma. J Clin Invest. (2024) 134:e178628. doi: 10.1172/jci178628 39352749 PMC11563674

[19] Liu X Guo Q Gao G Cao Z Guan Z Jia B . Exosome-transmitted circCABIN1 promotes temozolomide resistance in glioblastoma via sustaining ErbB downstream signaling. J Nanobiotechnol. (2023) 21:45. doi: 10.1186/s12951-023-01801-w 36755314 PMC9906870

[20] Song S Xie S Liu X Li S Wang L Jiang X . miR-3200 accelerates the growth of liver cancer cells by enhancing Rab7A. NCRNA. (2023) 8:675–85. doi: 10.1016/j.ncrna.2023.10.005 37860266 PMC10582768

[21] Mei H Zha Z Wang W Xie YS Huang YG Li WP . Surfaceome CRISPR screen identifies OLFML3 as a rhinovirus-inducible IFN antagonist (vol 22, 297, 2021). Genome Biol. (2021) 22:297. doi: 10.1186/s13059-021-02513-w 34686207 PMC8532573

[22] Yu Q Mei H Gu Q Zeng R Li Y Zhang J . OLFML3 promotes IRG1 mitochondrial localization and modulates mitochondrial function in macrophages. Int J Biol Sci. (2025) 21:2275–95. doi: 10.7150/ijbs.103859 40083707 PMC11900800

[23] Abdelmageed AA Ferran MC . The propagation, quantification, and storage of vesicular stomatitis virus. Curr Protoc Microbiol. (2020) 58:e110. doi: 10.1002/cpmc.110 32833351 PMC7449588

[24] Rehwinkel J Gack MU . RIG-I-like receptors: their regulation and roles in RNA sensing. Nat Rev Immunol. (2020) 20:537–51. doi: 10.1038/s41577-020-0288-3 32203325 PMC7094958

[25] Carpenter S O'Neill LAJ . From periphery to center stage: 50 years of advancements in innate immunity. Cell. (2024) 187:2030–51. doi: 10.1016/j.cell.2024.10.013 38670064 PMC11060700

[26] Hirschenberger M Hayn M Laliberté A Koepke L Kirchhoff F Sparrer KMJ . Luciferase reporter assays to monitor interferon signaling modulation by SARS-CoV-2 proteins. STAR Protoc. (2021) 2:100781. doi: 10.1016/j.xpro.2021.100781 34405154 PMC8361205

[27] Hu Z Xie Y Lu J Yang J Zhang J Jiang H . VANGL2 inhibits antiviral IFN-I signaling by targeting TBK1 for autophagic degradation. Sci Adv. (2023) 9:eadg2339. doi: 10.1126/sciadv.adg2339 37352355 PMC10289648

[28] Ozato K Shin D-M Chang T-H Morse HC . TRIM family proteins and their emerging roles in innate immunity. Nat Rev Immunol. (2008) 8:849–60. doi: 10.1038/nri2413 18836477 PMC3433745

[29] Huang Y Gao X He QY Liu W . A interacting model: How TRIM21 orchestrates with proteins in intracellular immunity. Small Methods. (2023) 28:e2301142. doi: 10.1002/smtd.202301142 37922533

[30] Li X Yang L Chen S Zheng J Zhang H Ren L . Multiple roles of TRIM21 in virus infection. Int J Mol Sci. (2023) 24:1683. doi: 10.3390/ijms24021683 36675197 PMC9867090

[31] Xue B Li H Guo M Wang J Xu Y Zou X . TRIM21 promotes innate immune response to RNA viral infection through Lys27-linked polyubiquitination of MAVS. J Virol. (2018) 92:e00321-18. doi: 10.1128/jvi.00321-18 29743353 PMC6026736

[32] Jones EL Laidlaw SM Dustin LB . TRIM21/Ro52 - Roles in innate immunity and autoimmune disease. Front Immunol. (2021) 12:738473. doi: 10.3389/fimmu.2021.738473 34552597 PMC8450407

[33] Jiang W Li X Xu H Gu X Li S Zhu L . UBL7 enhances antiviral innate immunity by promoting Lys27-linked polyubiquitination of MAVS. Cell Rep. (2023) 42:112272. doi: 10.1016/j.celrep.2023.112272 36943869

[34] Kong X Lu X Wang S Hao J Guo D Wu H . Type I interferon/STAT1 signaling regulates UBE2M-mediated antiviral innate immunity in a negative feedback manner. Cell Rep. (2023) 42:112002. doi: 10.1016/j.celrep.2023.112002 36662617

[35] Higgs R Lazzari E Wynne C Ní Gabhann J Espinosa A Wahren-Herlenius M . Self protection from anti-viral responses--Ro52 promotes degradation of the transcription factor IRF7 downstream of the viral Toll-Like receptors. PloS One. (2010) 5:e11776. doi: 10.1371/journal.pone.0011776 20668674 PMC2909902

[36] Higgs R Ní Gabhann J Ben Larbi N Breen EP Fitzgerald KA Jefferies CA . The E3 ubiquitin ligase Ro52 negatively regulates IFN-beta production post-pathogen recognition by polyubiquitin-mediated degradation of IRF3. J Immunol (Baltimore Md 1950). (2008) 181:1780–6. doi: 10.4049/jimmunol.181.3.1780 18641315 PMC2824853

[37] Zhang Z Bao M Lu N Weng L Yuan B Liu Y-J . The E3 ubiquitin ligase TRIM21 negatively regulates the innate immune response to intracellular double-stranded DNA. Nat Immunol. (2013) 14:172–8. doi: 10.1038/ni.2492 23222971 PMC3645272

[38] Zhang B Cai T He H Huang X Chen G Lai Y . TRIM21 promotes rabies virus production by degrading IRF7 through ubiquitination. Int J Mol Sci. (2023) 24:10892. doi: 10.3390/ijms241310892 37446070 PMC10341556

[39] Shi J Zhang Z Chen H-Y Yao Y Ke S Yu K . Targeting the TRIM21-PD-1 axis potentiates immune checkpoint blockade and CAR-T cell therapy. Mol Ther. (2025) 33:1073–90. doi: 10.1016/j.ymthe.2025.01.047 39905727 PMC11897759

[40] Pan JA Sun Y Jiang YP Bott AJ Jaber N Dou Z . TRIM21 ubiquitylates SQSTM1/p62 and suppresses protein sequestration to regulate redox homeostasis. Mol Cell. (2016) 61:720–33. doi: 10.1016/j.molcel.2016.03.015 26942676 PMC4779181

[41] Xie F Fan J-X Wang H Song Q-L Xu Y-S Lan Q-S . Macrophage TRIM21 knockout inhibits septic acute lung injury by downregulating autophagy regulator protein ubiquitination. Autophagy. (2025) 21:2650–69. doi: 10.1080/15548627.2025.2519063 40509575 PMC12758249

[42] Yang P Gao S Shen J Liu T Lu K Han X . TRIM21-mediated ubiquitination of SQSTM1/p62 abolishes its Ser403 phosphorylation and enhances palmitic acid cytotoxicity. Autophagy. (2025) 21:178–90. doi: 10.1080/15548627.2024.2394308 39172027 PMC11702951

[43] Yang C Wang Z Kang Y Yi Q Wang T Bai Y . Stress granule homeostasis is modulated by TRIM21-mediated ubiquitination of G3BP1 and autophagy-dependent elimination of stress granules. Autophagy. (2023) 19:1934–51. doi: 10.1080/15548627.2022.2164427 36692217 PMC10283440

[44] Porritt RA Hertzog PJ . Dynamic control of type I IFN signalling by an integrated network of negative regulators. Trends Immunol. (2015) 36:150–60. doi: 10.1016/j.it.2015.02.002 25725583

[45] Hertzog PJ Williams BRG . Fine tuning type I interferon responses. Cytokine Growth Factor Rev. (2013) 24:217–25. doi: 10.1016/j.cytogfr.2013.04.002 23711406

[46] Ivashkiv LB Donlin LT . Regulation of type I interferon responses. Nat Rev Immunol. (2014) 14:36–49. doi: 10.1038/nri3581 24362405 PMC4084561

[47] Liu W Yan M Liu Y Wang R Li C Deng C . Olfactomedin 4 down-regulates innate immunity against Helicobacter pylori infection. PNAS. (2010) 107:11056–61. doi: 10.1073/pnas.1001269107 20534456 PMC2890768

[48] Liu W Yan M Sugui JA Li H Xu C Joo J . Olfm4 deletion enhances defense against Staphylococcus aureus in chronic granulomatous disease. J Clin Invest. (2013) 123:3751–5. doi: 10.1172/jci68453 23908114 PMC3754258

[49] Gong F Li R Zheng X Chen W Zheng Y Yang Z . OLFM4 regulates lung epithelial cell function in sepsis-associated ARDS/ALI via LDHA-mediated NF-κB signaling. J Inflammation Res. (2021) 14:7035–51. doi: 10.2147/jir.s335915 34955649 PMC8694847

[50] Minamitani T Iwakiri D Takada K . Adenovirus virus-associated RNAs induce type I interferon expression through a RIG-I-mediated pathway. J Virol. (2011) 85:4035–40. doi: 10.1128/jvi.02160-10 21248047 PMC3126113

[51] Yamaguchi T Kawabata K Kouyama E Ishii KJ Katayama K Suzuki T . Induction of type I interferon by adenovirus-encoded small RNAs. PNSA. (2010) 107:17286–91. doi: 10.1073/pnas.1009823107 20855616 PMC2951419

[52] Naesens L Haerynck F Gack MU . The RNA polymerase III-RIG-I axis in antiviral immunity and inflammation. Trends Immunol. (2023) 44:435–49. doi: 10.1016/j.it.2023.04.002 37149405 PMC10461603

