# Supplementary material for: OLFML3 negatively regulates RIG-I signaling in RNA virus infection
Source: Front Immunol. 2026 Jun 10;17:1810852. doi: 10.3389/fimmu.2026.1810852 (PMC13290906; doi:10.3389/fimmu.2026.1810852)
Supplement: Supplementary file 1 [file DataSheet1.pdf]

Supplementary Material

figs. S1 to S7

Tables S1 to S9

Supplementary Figures

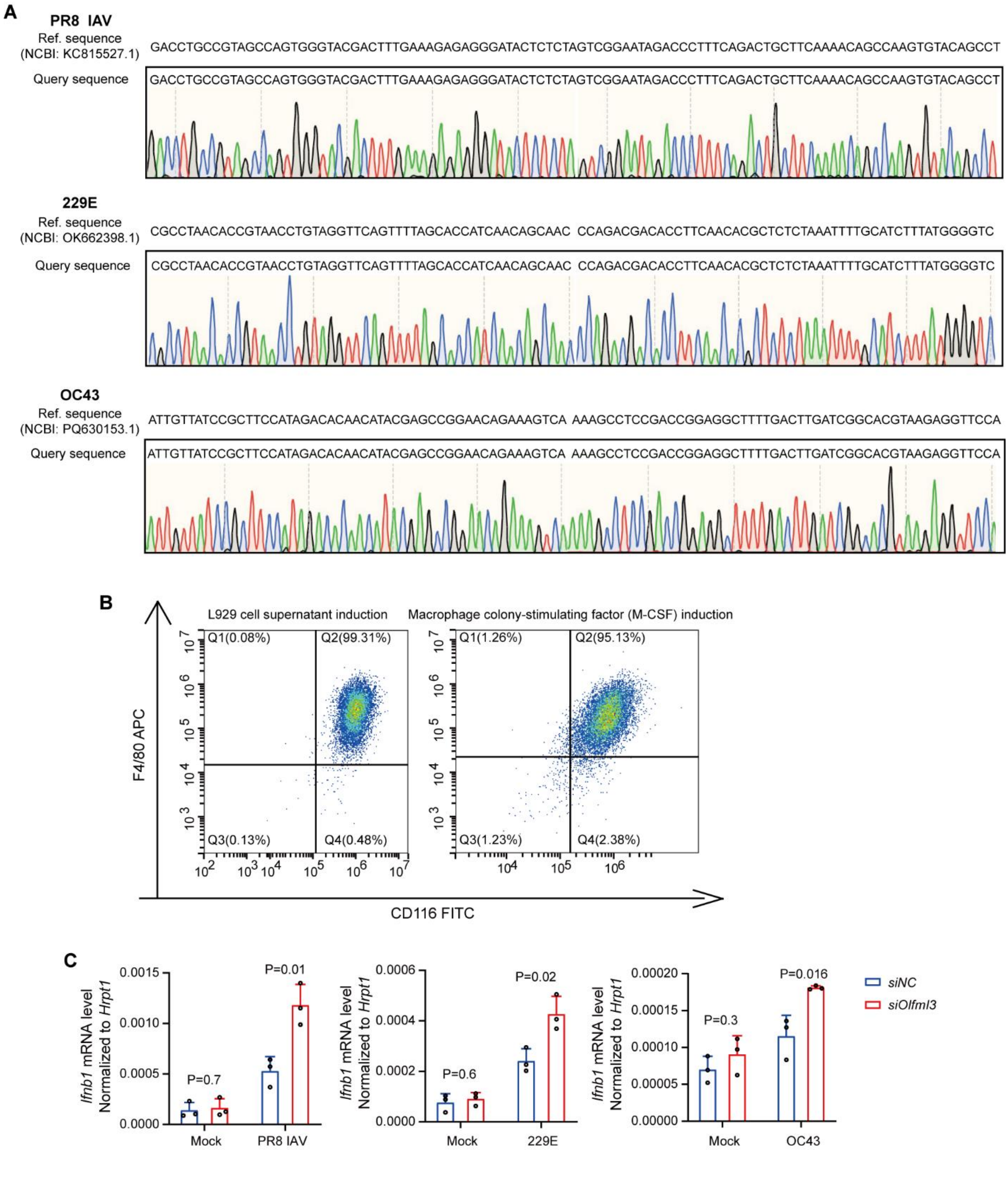

S1 Fig. Evaluation of the function of OLFML3 in RNA virus infection *in vitro*.

(**A**), Validation of the PR8 IAV, HCoV-229E and HCoV-OC43 viruses used in this study by comparing the Sanger sequenced nucleocapsid protein (NP) gene with the deposited sequences in NCBI database. (**B**), Flow cytometry analysis of the surface marker of primary mouse BMDMs. BMDMs are isolated from

mouse tibia and femur and stimulated with L929 cell supernatant or macrophage colony-stimulating factor (M-CSF). L929 cell supernatant leads to higher purity of BMDMs and is selected as the finalized procedure. (**C**), RT-qPCR analysis of the effects of *Olfml3* knockdown on IFN-β mRNA expression in mouse MLE-12 cells infected with PR8 IAV, HCoV-229E and HCoV-OC43. The cells are infected with viruses at an MOI of 1.0 for 24 h. Mock, medium treatment without viruses. siNC, negative control with non-targeting siRNA. The data are from three independent biological replicates and presented as mean ± SD. The significant difference is analyzed by two-tailed unpaired Student's t test unless noted otherwise. Source data are provided as a Source Data file.

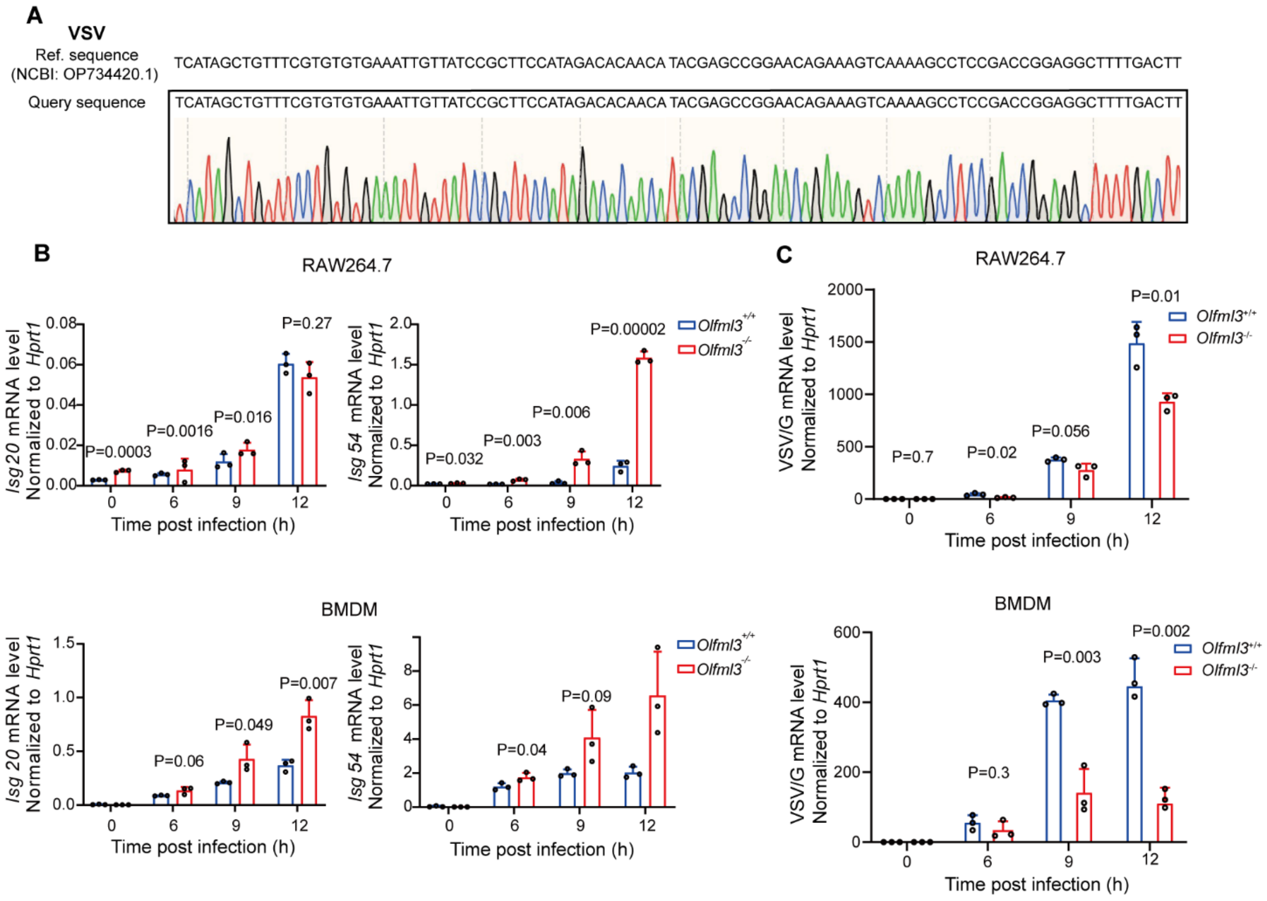

S2 Fig. The effects of OLFML3 on VSV-induced IFN production *in vitro*.

(**A**), Validation of the VSV virus used in this study by comparing the Sanger sequenced glycoprotein (G) gene with the deposited sequences in NCBI database. (**B**), RT-qPCR analysis of the effects of *Olfml3* knockout on VSV-induced ISG mRNA expression in RAW264.7 cells and isolated BMDMs. (**C**), RT-qPCR analysis of VSV-G mRNA expression in *Olfml3*$^{+/+}$ and *Olfml3*$^{-/-}$ RAW246.7 cells and BMDMs. Cells are infected with VSV at a multiplicity of infection (MOI) of 0.3 and the samples collected at indicated time points. For (B-C), the data are from three independent biological replicates and presented as mean ± SD. The significant difference is analyzed by two-tailed unpaired Student's t test. Source data are provided as a Source Data file.

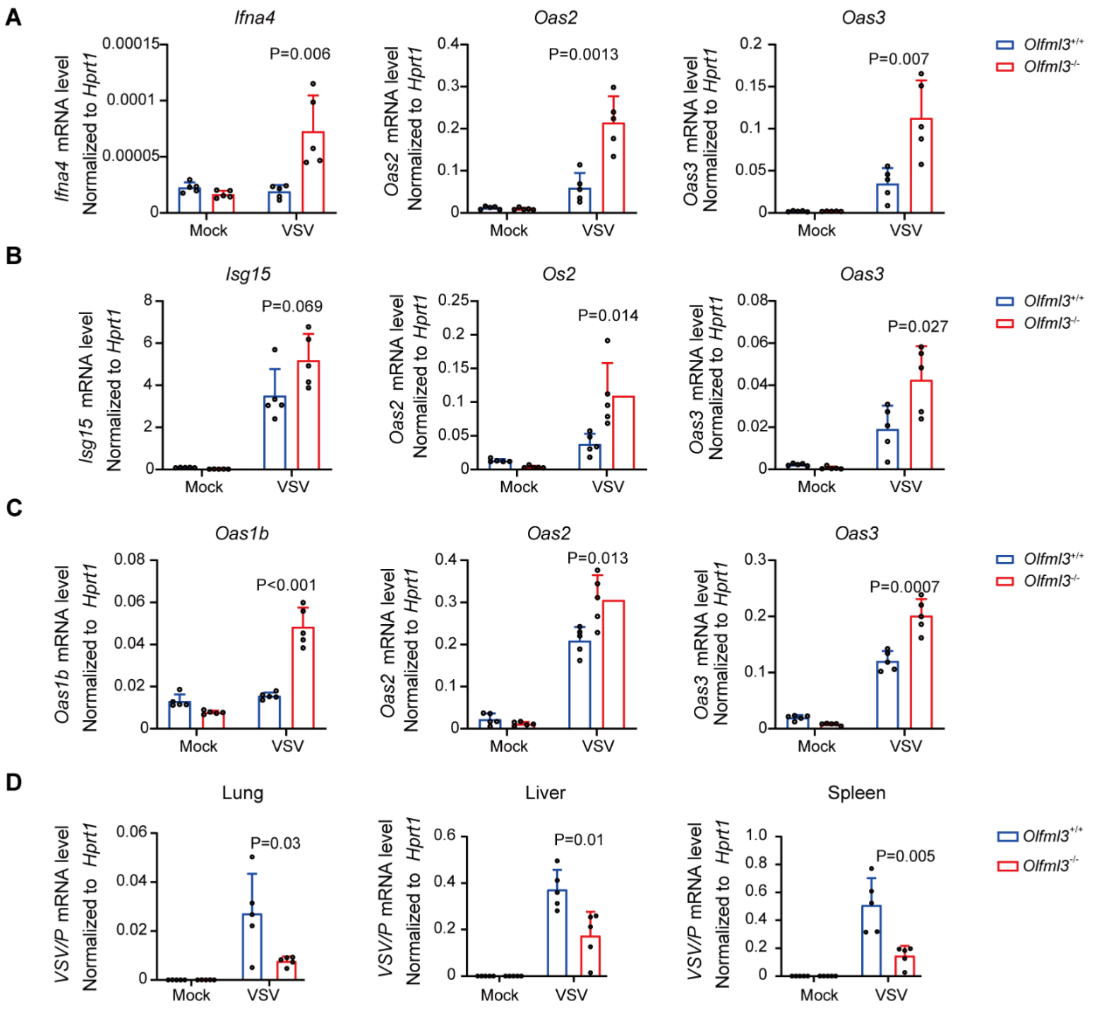

S3 Fig. The effects of *Olfml3* depletion on VSV infection.

(**A-C**), RT-qPCR analysis of ISG mRNA expression in the lung (A), liver (B) and spleen (C) of *Olfml3*$^{+/+}$ and *Olfml3*$^{-/-}$ mice (n = 5 per group). (**D**), RT-qPCR analysis of VSV-G mRNA expression in the lung, liver and spleen of *Olfml3*$^{+/+}$ and *Olfml3*$^{-/-}$ mice (n = 5 per group). VSV is administrated via intraperitoneal injection at a dosage of 1.5×10^6 PFU per gram body weight and the samples are collected at 24 h post infection. The data are from five independent biological replicates and presented as mean ± SD. The significant difference is analyzed by two-tailed unpaired Student's t test. Mock, DMEM treatment without VSV infection. Source data are provided as a Source Data file.

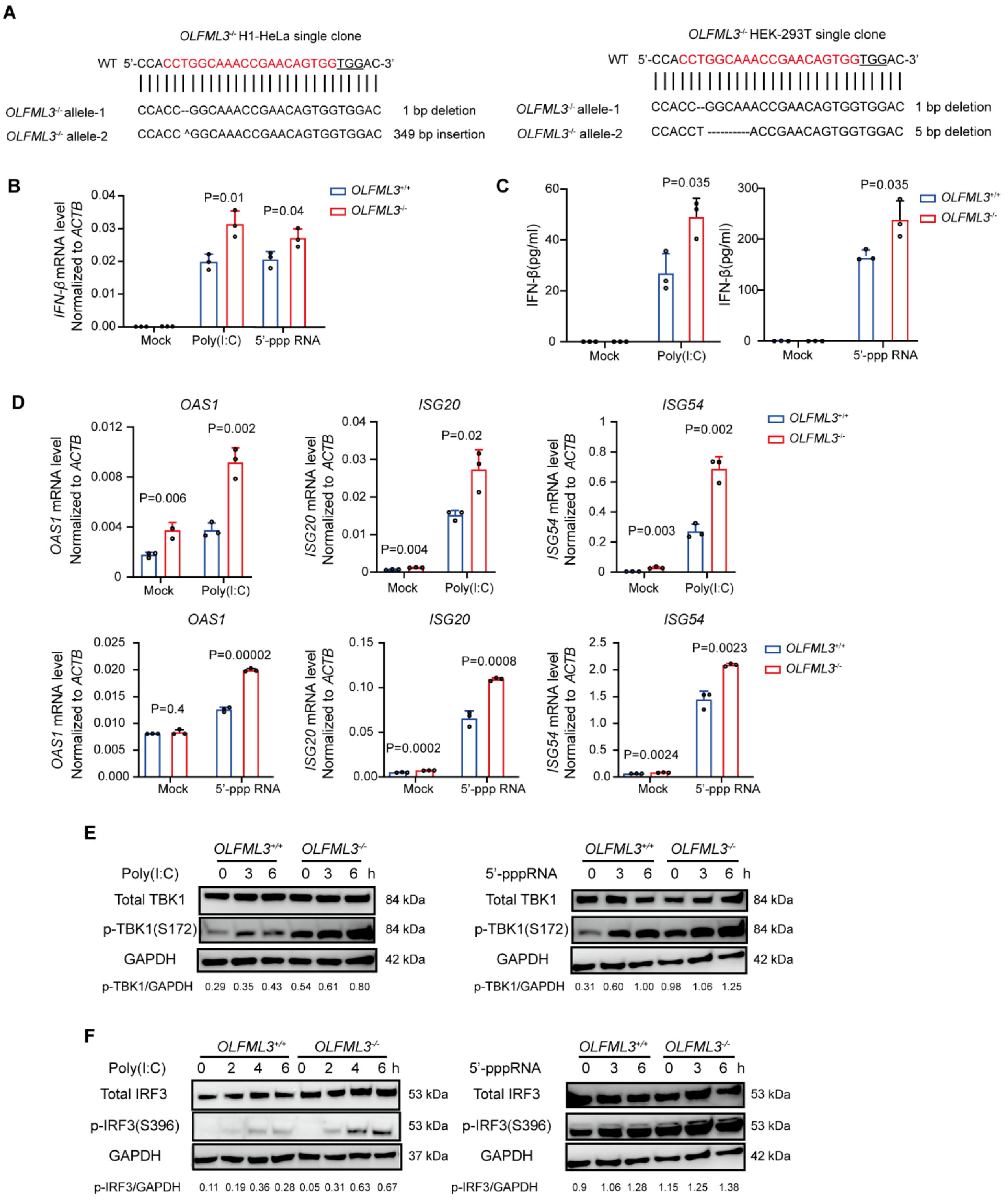

S4 Fig. Evaluation of the effects of OLFML3 on vRNA mimic-induced IFN and ISG production in H1-HeLa cells.

(**A**), Characterization of *OLFML3*$^{-/-}$ single clones of H1-HeLa and HEK-293T by Sanger sequencing analysis of mutated alleles. The 20-bp CRISPR-Cas9 targeting sequence is highlighted in red and

protospacer adjacent motif (PAM) underlined. (**B-C**), The effects of *OLFML3* knockout on Poly(I:C)- or 5'-ppp RNA-induced IFN-β mRNA(B) and protein (C) production in H1-HeLa, as determined by RT-qPCR or ELISA analyses. (**D**), RT-qPCR analysis of the effects of *OLFML3* knockout on Poly(I:C)- or 5'-ppp RNA-induced ISG mRNA expression in H1-HeLa cells. For (B-D), The cells are transfected with Poly(I:C) and 5'-ppp RNA and the samples are collected at 9 h post transfection. The data are from three independent biological replicates and presented as mean ± SD. The significant difference is analyzed by two-tailed unpaired Student's t test. (**E-F**), WB analysis of the effects of *OLFML3* knockout on vRNA-induced TBK1 phosphorylation (E) and IRF3 phosphorylation (F) in H1-HeLa cells. The cells are transfected with Poly(I:C) or 5'-ppp RNA and the samples are collected at indicated time points. Source data are provided as a Source Data file.

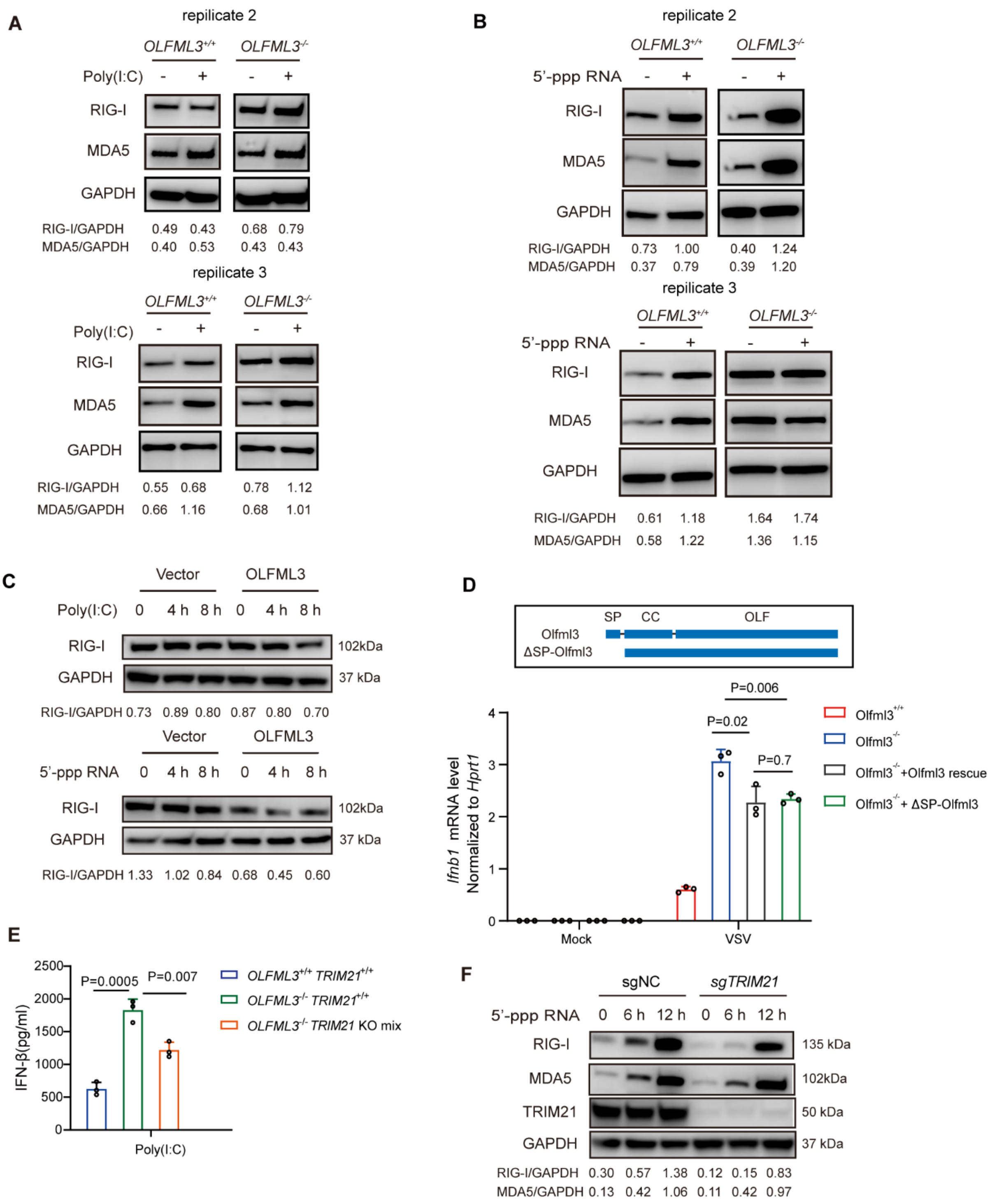

S5 Fig. OLFML3 destabilized RIG-I like receptors in a TRIM21-dependent manner.

(A-B), Two additional biological replicates for the WB analysis of the effects of *OLFML3* knockout on RLRs in H1-HeLa cells upon Poly(I:C) (**A**) and 5'-ppp RNA stimulation (**B**) (related to main text Fig. 4A-B). The images in panel B are from the same gel slice and the middle lanes not related to this experiment are removed. (**C**), WB analysis of the effects of *OLFML3* overexpression on *OLFML3*$^{-/-}$ H1-HeLa cells. For **A-C**, the cells are transfected with empty vector (Vector) or OLFML3, followed by treatment with

Poly(I:C) or 5'-ppp RNA. The samples are collected at 6 h after transfection. (**D**), RT-qPCR analysis of the effects of *Olfml3* knockout, Olfml3 rescue and ΔSP-Olfml3 rescue on VSV-induced *Ifnb1* mRNA expression in RAW264.7 cells. (**E**), ELISA analysis of the effect of *TRIM21* knockout on IFN-β protein secretion in *OLFML3*$^{+/+}$ and *OLFML3*$^{-/-}$ H1-HeLa cells. The cells are treated with Poly(I:C) for 9 h. (**F**), WB analysis of the effects of *TRIM21* knockout on RIG-I and MDA5 in H1-HeLa cells. The cells are treated with 5'-pppRNA for indicated time. sg, mixed population of knockout cells without isolation of single clones induced by sgRNA transduction.

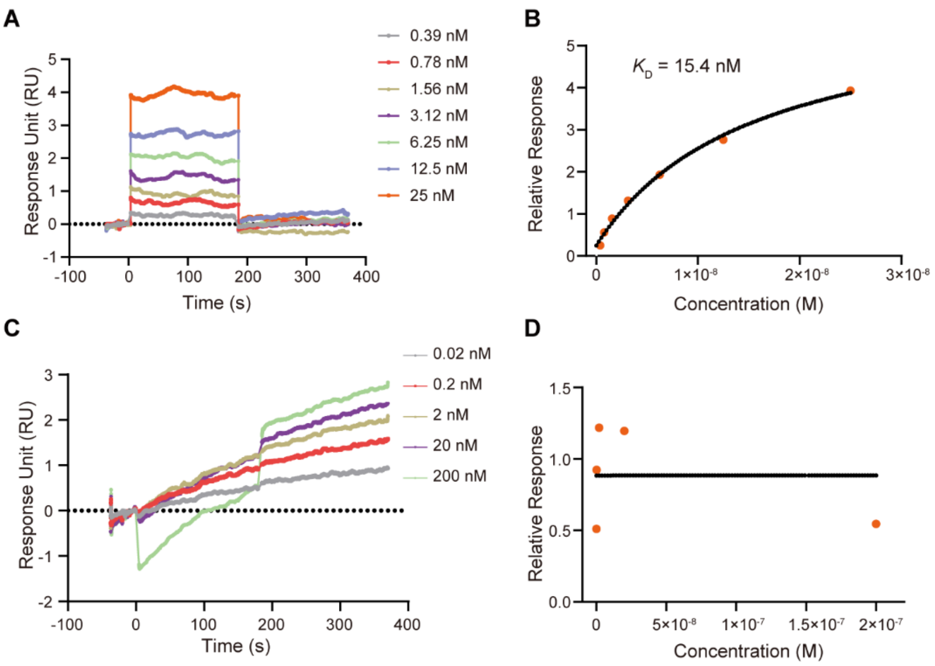

S6 Fig. Analysis of the interaction between OLFML3 (ligand) and anti-OLFML3 antibody or TRIM21 (analytes) using SPR with Biacore 8K.

(**A**), Association and dissociation curves for OLFML3 and anti-OLFML3 antibody binding. (**B**) Fitting of the binding curve between OLFML3 and anti-OLFML3 antibody using a steady-state affinity method. (**C**) Association and dissociation curves for OLFML3 and TRIM21 binding. (**D**) Fitting of the binding curve between OLFML3 and TRIM21 using a steady-state affinity method.

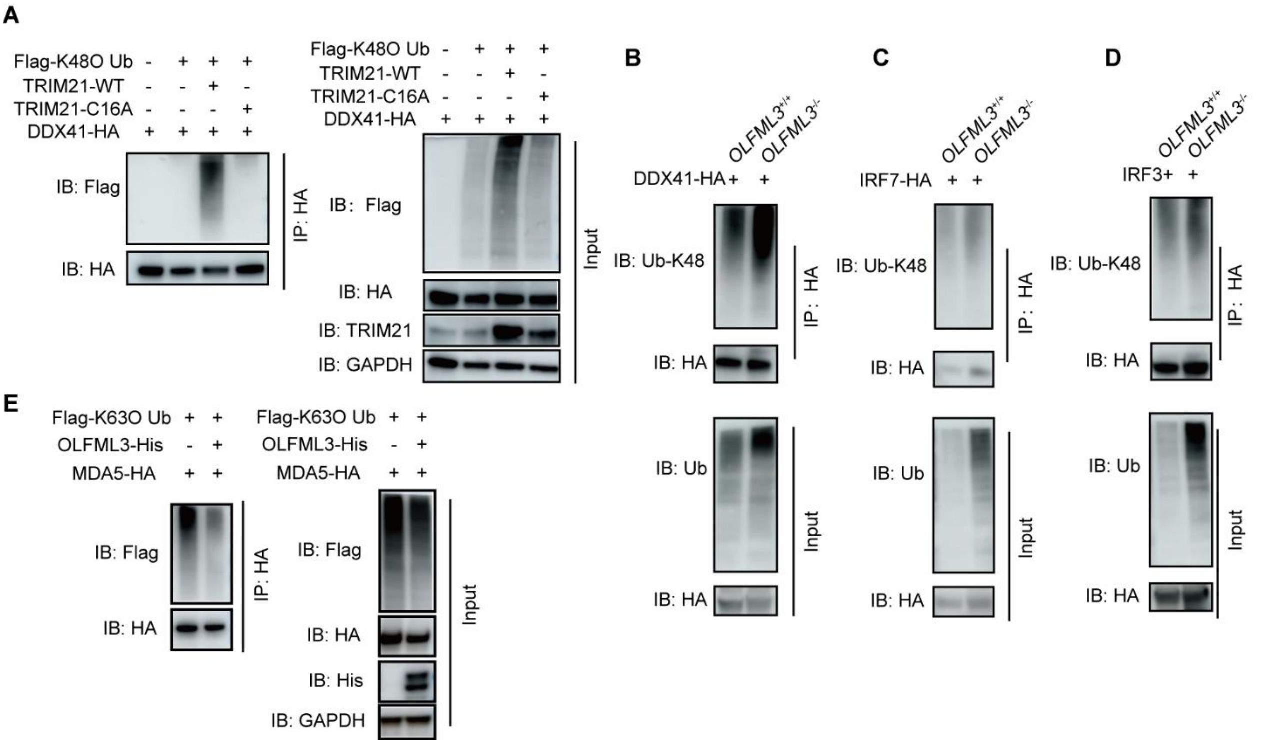

S7 Fig. OLFML3 inhibits TRIM21 activity.

(**A**), Co-IP analysis of the effects of TRIM21 wide type or C16A mutant on RIG ubiquitination in HEK-293T cells. K48O Ub, ubiquitin with the only lysine at position 48. (**B-D**), Co-IP analysis of K48 ubiquitination of DDX41 (B), IRF7 (C) and IRF3 (D) in *OLFML3*$^{+/+}$ and *OLFML3*$^{-/-}$ HEK-293T cells. (**E**), Co-IP analysis of the effects of *OLFML3* overexpression on MDA5 ubiquitination in HEK-293T cells. K63O Ub, ubiquitin with only one lysine residue at position 63.

Supplementary Tables

S1 Table. Primers for genotyping of *Olfml3*$^{-/-}$ mice

	Forward primers	Reverse primers
PCR-1	aggcaatctgcctgcaacttcc	gcactgttactgagcttgcatatgttg
PCR-2	gagggcaagacctgtttatctggt	aggagagatgccgcagcagttag

S2 Table. Primers for construction of sgRNA plasmid

Genes	Forward primers	Reverse primers
Nontarget sgRNA	caccgacggaggctaagcgtcgcaa	aaacttgcgacgcttagcctccgtc
OLFML3 (human)	caccgcctggcaaaccgaacagtggt	aaacaccactgttcggtttgccagg
Olfml3 (mouse)	caccgatgtcccagagagaacgcagagg	aaaccctctgcgttctctctgggacat
TRIM21 (human)	caccggagcctgtgagcatcgagtg	aaaccactcgatgctcacaggctc

S3 Table. Primers for PCR amplification of sgRNA targeted sites for Sanger sequencing analyses

Genes	Forward primers	Reverse primers
OLFML3 (human)	gtggtacccgctctttctcaa	aaataagggagtgctgcccg
TRIM21 (human)	cggggtacccctgtaaagccaaaccccct	ccgctcgagctgctaaagctcgcttgctg

S4 Table. Primers for qPCR

Genes	Forward primers	Reverse primers
Hprt1 (mouse)	tcagtcaacgggggacataaa	ggggctgtactgcttaaccag
Ifn-β (mouse)	cagctccaagaaaggacgaac	ggcagtgtaactcttctgcat
Isg20 (mouse)	tgggcctcaaagggtgagt	cgggtcggatgtacttgtcata
Isg54 (mouse)	aagcacctcaaagggcaaaac	tcggcccatgtgatagtagac
Oas1 (mouse)	gggcctctaaaggggtcaag	tcaaacttcactccacaacgtc
Olfml3 (mouse)	caccttgtggagtacatggaac	ctacctccctttcaagacggt
β-actin (human)	catgtacgttgctatccaggc	ctccttaatgtcacgcacgat
IFN-β (human)	gacatccctgaggagattaag	atgttctggagcatctcatag
ISG20 (human)	ctcgttgcagcctcgtgaa	cgggttctgtaatcggtgatctc
OAS1 (human)	tgtccaaggtggtaaagggtg	ccggcgatttaactgatcctg
TRIM21 (human)	tcagcagcacgcttgacaat	ggccacactcgatgctcac
ISG54 (human)	ggagagcaatctgcgacag	gctgcctcatttagacctctg
OLFML3 (human)	tccttttgtcatggtcgggac	taaagcagctagtcggcgttc
VSVG	caagtcaaaatgcccaagagtcaca	tttccttgcattgttctacagatgg
VSVP	caagtcaaaatgcccaagagtcaca	tttccttgcattgttctacagatgg
PR8-NP	tgtgtatggacctgccgtagc	ccatccacaccagttgactcttg
229E-NP	ggcaaacgggtggatttgtc	cgcctaacaccgtaacctgt
OC43-NP	agcaaccaggctgatgtcaatacc	agcagaccttcctgagccttcaat

S5 Table. Human codon-optimized OLFML3 gene for overexpression experiments

OLFML3-Myc-Flag (ORF)
atgggccctagcacgccattactgatcctgttcctgctgagctggagcggccctctgcagggccagcagcaccacctggtggagtacatggagaga agactggccgccctggaggagagactggcccagtgccaggaccagagcagcagacacgccgccgagttaagggatttcaagaataaaatgttgc ctctgctggaggtggccgagaaggagagagaggccctgagaaccgaggccgacaccatcagcggcagagtggacagactggagagagaggtg gactacctggagacccagaatcctgccctgccttgcgtggagttcgacgagaaggtgaccggcggccctggcaccaagggcaagggcagaagaa atgagaagtacgacatggtgaccgactgcggctacaccatcagccaggtgagaagcatgaagatcctgaagagattcggcggccctgccggcctg tggaccaaggaccctctgggccagaccgagaagatctacgtgctggacggcacccagaatgacaccgccttcgtgttccctagattgcgagatttca ctttagccatggccgccagaaaggccagcagagtgagagtgcctttcccttgggtgggcaccggccagctggtgtacggcggcttcctgtacttcgc cagaagacctcctggcagacctggcggcggcggcgagatggagaataccctgcagctgatcaagttccatctggccaatagaaccgtggtagaca gcagcgtgttccctgccgagggcctgatccctccttacggcctgaccgccgacacctacatcgacctggccgccgacgaggagggcctgtgggcc gtgtacgccaccagagaggacgacagacacctgtgcctggccaagctggaccctcagaccctggacaccgagcagcagtgggacactccgtgtc ctagagagaatgccgaggccgccttcgtgatctgcggcaccctgtacgtggtgtacaataccagacctgccagcagagccagaatccagtgcagct tcgacgccagcggcaccctgactcccgaaagagccgccctgccttacttccctagaagatacggcgcccacgccagcctgagatacaatcctagag agagacagctgtacgcctgggacgacggctaccagatcgtgtacaagctggagatgagaaagaaggaggaggaggtgacgcgtacgcggccgg agcagaaactcatctcagaagaggatctggcagcaaatgatatcctggattacaaggatgacgacgataaggtttaa

S6 Table. siRNA sequences

Genes	Sense sequences (5’-3’)	Anti-sense sequences (5’-3’)
Nontarget	cagcacgcuugacaaugautt	aucauugucaagcgugcugtt
TRIM21-siRNA	gcagagcauaccuggaaautt	auuuccagguaugcucugctt

S7 Table. Proteins list identified by mass spectrometry

Proteins Co-IP with empty Vector	Proteins Co-IP with OLFML3-Myc
40S ribosomal protein S18	Olfactomedin-like protein 3
40S ribosomal protein S7	Heat shock cognate 71 kDa protein
60S ribosomal protein L23a	40S ribosomal protein S18
40S ribosomal protein S25	Putative RNA-binding protein Luc7-like 2
Immunoglobulin heavy variable 3-33	40S ribosomal protein S4, X isoform
60S ribosomal protein L11	40S ribosomal protein S3
40S ribosomal protein S3	Fibronectin
Putative RNA-binding protein Luc7-like 2	40S ribosomal protein S7
Immunoglobulin heavy variable 1-45	Tubulin beta chain
Fibrinogen gamma chain	Elongation factor 1-alpha 1
Plectin	Splicing factor U2AF 65 kDa subunit
Pre-rRNA-processing protein TSR1 homolog	40S ribosomal protein S16
Immunoglobulin heavy variable 3-43	RNA-binding protein 39
40S ribosomal protein S14	Ubiquitin-40S ribosomal protein S27a
POTE ankyrin domain family member F	40S ribosomal protein S25
40S ribosomal protein S4, X isoform	60S ribosomal protein L23
Isocitrate dehydrogenase [NADP] cytoplasmic	Pre-rRNA-processing protein TSR1 homolog
60S ribosomal protein L29	60S ribosomal protein L23a
Tubulin alpha-1B chain	60S ribosomal protein L11
40S ribosomal protein S30	Fibrinogen beta chain
Fibrinogen beta chain	Fibrinogen gamma chain
Splicing factor U2AF 65 kDa subunit	40S ribosomal protein S3a
Cleavage and polyadenylation specificity factor subunit 6	Myosin light polypeptide 6
Immunoglobulin heavy variable 3-11	Immunoglobulin heavy variable 3-30
40S ribosomal protein S16	Histone H4
Fibronectin	Splicing factor U2AF 35 kDa subunit
Splicing factor U2AF 35 kDa subunit	40S ribosomal protein S11
Elongation factor 1-alpha 2	Histone H2A type 2-A
Histone H4	Desmoplakin
60S ribosomal protein L38	40S ribosomal protein S17
ATP synthase subunit beta, mitochondrial	Immunoglobulin heavy variable 1-45
Small nuclear ribonucleoprotein Sm D2	Cleavage and polyadenylation specificity factor subunit 6
RNA-binding protein 39	40S ribosomal protein S14
Desmoplakin	GTP-binding nuclear protein Ran
60S ribosomal protein L35	ATP synthase subunit beta, mitochondrial
Negative elongation factor B	40S ribosomal protein S15a
Immunoglobulin heavy constant gamma 2	40S ribosomal protein S8
Immunoglobulin kappa variable 4-1	40S ribosomal protein S5

ATP-dependent RNA helicase DHX29	Heat shock protein HSP 90-alpha
40S ribosomal protein S6	60S ribosomal protein L9
Histone H1.3	60S ribosomal protein L35
Activated RNA polymerase II transcriptional coactivator p15	Plectin
40S ribosomal protein S17	Peroxiredoxin-1
Filamin A-interacting protein 1-like	ADP/ATP translocase 2
ATP-binding cassette sub-family B member 9	BAG family molecular chaperone regulator 2
Putative uncharacterized protein C15orf56	Histone H2B type 1-A
	Histone H1.3
	Luc7-like protein 3
	Calnexin
	60S ribosomal protein L13a
	Immunoglobulin kappa variable 4-1
	E3 ubiquitin-protein ligase TRIM21
	40S ribosomal protein S30
	60S ribosomal protein L29
	Immunoglobulin heavy constant gamma 2
	Activated RNA polymerase II transcriptional coactivator p15
	40S ribosomal protein S20
	Small nuclear ribonucleoprotein Sm D2

S8 Table. DNA sequences of ubiquitin genes for overexpression experiments

**Flag-WT-Ubiquitin (ORF)**
atggattacaaggacgacgatgacaagtccggactcagatctcgagctcaagcttcgaattctatgcagatcttcgtgaaaacccttaccggcaagaccatcacccttgaggtggagcccagtgacaccatcgaaaatgtgaaggccaagatccaggataaggaaggcattcccccgaccagcagaggctcatctttgcaggcaagcagctggaagatggccgtactctttctgactacaacatccagaaggagtcgaccctgcacctggtcctgcgtctgagaggtggttaa
**Flag-K48O-ubiquitin (ORF)**
atggattacaaggacgacgatgacaagtccggactcagatctcgagctcaagcttcgaattctatgcagatcttcgtgcgcacccttaccggccgcaccatcacccttgaggtggagcccagtgacaccatcgaaaatgtgcgcgcccgcatccaggatcgcgaaggcattcccccgaccagcagaggctcatctttgcaggcaagcagctggaagatggccgtactctttctgactacaacatccagcgcgagtcgaccctgcacctggtcctgcgtctgagaggtggttaa
**Flag-K63O-ubiquitin (ORF)**
atggattacaaggacgacgatgacaagtccggactcagatctcgagctcaagcttcgaattctatgcagatcttcgtgcgcacccttaccggccgcaccatcacccttgaggtggagcccagtgacaccatcgaaaatgtgcgcgcccgcatccaggatcgcgaaggcattcccccgaccagcagaggctcatctttgcaggccgccagctggaagatggccgtactctttctgactacaacatccagaaggagtcgaccctgcacctggtcctgcgtctgagaggtggttaa

S9 Table. DNA sequences of RIG-I genes for overexpression experiments

**RIG-I-HA (ORF)**
atgaccaccgagcagcgacgcagcctgcaagccttccaggattatatccggaagaccctggaccctacctacatcctgagctacatggccccctggt<br>ttagggaggaagaggtgcagtatattcaggctgagaaaaacaacaagggcccaatggaggctgccacactttttctcaagttcctgttggagctccag<br>gaggaaggctggttccgtggctttttggatgccctagaccatgcaggttattctggactttatgaagccattgaaagttgggatttcaaaaaaattgaaaa<br>gttggaggagtatagattacttttaaaacgtttacaaccagaatttaaaaccagaattatcccaaccgatatcatttctgatctgtctgaatgtttaattaatca<br>ggaatgtgaagaaattctacagatttgctctactaaggggatgatggcaggtgcagagaaattggtggaatgccttctcagatcagacaaggaaaact<br>ggcccaaaactttgaaacttgctttggagaaagaaaggaacaagttcagtgaactgtggattgtagagaaaggtataaaagatgttgaaacagaagat<br>cttgaggataagatggaaacttctgacatacagattttctaccaagaagatccagaatgccagaatcttagtgagaattcatgtccaccttcagaagtgtc<br>tgatacaaacttgtacagcccatttaaaccaagaaattaccaattagagcttgctttgcctgctatgaaaggaaaaaacacaataatatgtgctcctacag<br>gttgtggaaaaacctttgtttcactgcttatatgtgaacatcatcttaaaaaattcccacaaggacaaaaggggaaagttgtctttttgcgaatcagatccc<br>agtgtatgaacagcagaaatctgtattctcaaaatactttgaaagacatgggtatagagttacaggcatttctggagcaacagctgagaatgtcccagtg<br>gaacagattgttgagaacaatgacatcatcattttaactccacagattcttgtgaacaaccttaaaaagggaacgattccatcactatccatctttactttga<br>tgatatttgatgaatgccacaacactagtaaacaacacccgtacaatatgatcatgtttaattatctagatcagaaacttggaggatcttcaggcccactg<br>ccccaggtcattgggctgactgcctcggttggtgttggggatgccaaaaacacagatgaagccttggattatatctgcaagctgtgtgcttctcttgatg<br>cgtcagtgatagcaacagtcaaacacaatctggaggaactggagcaagttgtttataagccccagaagtttttcaggaaagtggaatcacggattagc<br>gacaaatttaaatacatcatagctcagctgatgagggacacagagagtctggcaaagagaatctgcaaagacctcgaaaacttatctcaaattcaaaat<br>agggaatttggaacacagaaatatgaacaatggattgttacagttcagaaagcatgcatggtgttccagatgccagacaaagatgaagagagcagga<br>tttgtaaagccctgtttttatacacttcacatttgcggaaatataatgatgccctcattatcagtgagcatgcacgaatgaaagatgctctggattacttgaa<br>agacttcttcagcaatgtccgagcagcaggattcgatgagattgagcaagatcttactcagagatttgaagaaaagctgcaggaactagaaagtgtttc<br>cagggatcccagcaatgagaatcctaaacttgaagacctctgcttcatcttacaagaagagtaccacttaaacccagagacaataacaattctctttgtg<br>aaaaccagagcacttgtggacgctttaaaaaattggattgaaggaaatcctaaactcagttttctaaaacctggcatattgactggacgtggcaaaacaa<br>atcagaacacaggaatgaccctcccggcacagaagtgtatattggatgcattcaaagccagtggagatcacaatattctgattgccacctcagttgctg<br>atgaaggcattgacattgcacagtgcaatcttgtcatcctttatgagtatgtgggcaatgtcatcaaaatgatccaaaccagaggcagaggaagagcaa<br>gaggtagcaagtgcttccttctgactagtaatgctggtgtaattgaaaaagaacaaataaacatgtacaaagaaaaaatgatgaatgactctattttacgc<br>cttcagacatgggacgaagcagtatttagggaaaagattctgcatatacagactcatgaaaaattcatcagagatagtcaagaaaaaccaaaacctgta<br>cctgataaggaaaataaaaaactgctctgcagaaagtgcaaagccttggcatgttacacagctgacgtaagagtgatagaggaatgccattacactgt<br>gcttggagatgcttttaaggaatgctttgtgagtagaccacatcccaagccaaagcagttttcaagttttgaaaaaagagcaaagatattctgtgcccga<br>cagaactgcagccatgactggggaatccatgtgaagtacaagacatttgagattccagttataaaaattgaaagttttgtggtggaggatattgcaactg<br>gagttcagacactgtactcgaagtggaaggactttcattttgagaagataccatttgatccagcagaaatgtccaaactcgagtacccatacgacgtcc<br>cagactacgcttga
